# Electrostatic Charge-Based Online Monitoring of the Grinding Process

**DOI:** 10.3390/s26175449

**Published:** 2026-08-28

**Authors:** Pengtao Li, Xiaofei Duan, Xiang Zhang, Hongfu Zuo, Qi Hua, Yongwei Liu

**Affiliations:** 1 School of Mechanics and Safety Engineering, Zhengzhou University, Zhengzhou 450001, China; zhangxiang@zzu.edu.cn; 2 State-Owned Luoyang Dancheng Radio Factory, Luoyang 471000, China; duanxiaofeix@163.com (X.D.); huaqi071@163.com (Q.H.); lywcand81@163.com (Y.L.); 3 College of Civil Aviation, Nanjing University of Aeronautics and Astronautics, Nanjing 211100, China; rms@nuaa.edu.cn

**Keywords:** grinding, online monitoring, static electricity, grinding force, wear state

## Abstract

Reliable online condition monitoring is essential for maintaining process stability and guaranteeing machining quality in precision grinding. Against this background, this study proposes an electrostatic induction-based measurement strategy and further performs systematic comparisons with conventional force-based monitoring methods. First, an electrostatic sensor model is established to quantitatively characterize charge transfer behaviors during grinding interactions. A three-axis experimental grinding platform is deployed to correlate electrostatic responses and mechanical force signals with critical grinding variables, including grinding speed, depth of cut, feed rate, and wheel wear severity. To achieve quantitative and objective evaluation, a unit-free Dynamic Sensitivity Change Degree (DSCD) index is introduced. Comparative results based on the DSCD index reveal that electrostatic signals exhibit better performance than traditional force-based signals in tracking grinding wheel speed variations and progressive wear evolution under the tested conditions. Meanwhile, the DSCD fluctuation in electrostatic signals remains within 1% under varying cutting depths, indicating favorable linear stability within the scope of the present experiments. Furthermore, a Generalized Conditional Variational Auto-Encoder (G-CVAE) model is developed to augment insufficient wheel wear datasets. The generated synthetic signals exhibit high fidelity and enable accurate and robust classification of grinding wheel wear states. This study verifies the existence of explicit quantitative correlations between electrostatic induction signals and key grinding parameters as well as wheel wear conditions. The proposed monitoring method can provide high-quality, reliable data support for subsequent grinding condition assessment and intelligent process decision-making.

## 1. Introduction

Grinding represents a pivotal precision machining process widely employed in modern mechanical manufacturing. Real-time online monitoring of grinding process parameters is essential to dynamically adjust machining conditions, inhibit grinding burn and abrasive bluntness, and secure the dimensional accuracy and surface integrity of machined workpieces [1]. A variety of sensing technologies have been developed for grinding condition monitoring, and mainstream measurement approaches can be classified into grinding force measurement, grinding temperature measurement, acoustic emission (AE) sensing, vibration sensing, and spindle power monitoring. Each sensing methodology faces distinct practical limitations when deployed in industrial scenarios.

Grinding force monitoring is the most mature in-process measurement technique applied on grinding production lines [2,3,4]. By mounting multi-component dynamometers underneath worktables or spindles, researchers can quantitatively correlate normal and tangential grinding forces with material removal behavior to regulate machining quality. To improve the feature extraction accuracy of force signals, Yang et al. [5] adopted one-dimensional convolutional neural networks to excavate multivariate features from force–time series for abrasive wheel wear prediction. Nevertheless, commercial dynamometers suffer from two prominent drawbacks: high procurement costs and stringent spatial installation constraints, which greatly hinder the large-scale deployment of force-based monitoring in automated mass production.

Grinding temperature monitoring, a popular research direction for preventing thermal damage, can be categorized into direct contact measurement and indirect non-contact measurement [6]. Direct measurement generally adopts embedded thermocouples to capture transient temperatures within the contact zone between the grinding wheel and workpiece [7]. However, embedding thermocouple wires destroys workpiece surface integrity and cannot be reused in batch machining, restricting its industrial promotion. Indirect temperature monitoring mainly relies on infrared thermal imagers to acquire surface temperature fields of machined components [8]. Vedhavalli et al. [9] proposed a novel indirect prediction approach and revealed that air oxidation reduces the carbon content of grinding chips, and the chip carbon concentration exhibits a close correlation with grinding temperature. Although chip carbon measurement offers an alternative route for indirect thermal monitoring, rigorous quantitative mapping between chip carbon characteristics and practical machining quality indicators remains absent, making closed-loop parameter adjustment infeasible.

Grinding vibration monitoring commonly employs acoustic emission (AE) sensors to capture transient elastic waves induced by abrasive friction, material micro-fracture and microcrack initiation on grinding wheels [10,11]. Raw AE signals have two notable shortcomings: cumbersome signal processing and feature extraction hinders industrial applications while mandatory contact installation weakens monitoring flexibility in high-speed automated grinding equipment. Scholars have continuously optimized AE-based detection schemes. Yesilyurt et al. [12] adopted scalogram instantaneous features to detect grinding burn and verified that scalogram phase deviation acts as a reliable indicator of thermal damage. Li et al. [13] integrated deep learning with AE signal decomposition to extract high-dimensional features for tracking progressive wheel wear, overcoming the weak discrimination capacity of conventional time–frequency features. Duan et al. [14] fused AE and grinding force signals to improve wear recognition accuracy; however, deploying multiple contact sensors increases hardware costs and layout complexity. To identify the wear states of abrasive belts, Liu et al. [15] constructed a mechanism-informed acoustic-force fusion framework and adopted stacking ensemble learning to realize decision-level information fusion, achieving an average recognition accuracy of 97.93%. Chen et al. [16] proposed a neural network-based method for abrasive belt wear monitoring with enhanced acoustic features, which yields average accuracy, precision, recall and f1-score of 92.46%, 91.90%, 91.67% and 0.914, respectively. This approach provides technical support for intelligent maintenance of grinding systems. A 2025 investigation into CBN grinding of precision bearing raceways further exposed the limitations of contact AE sensors [17]: severe industrial noise contaminates raw signals, and complicated decomposition algorithms raise the barriers for on-site implementation. Electromechanical impedance (EMI) measurement has emerged as a non-contact alternative to remove contact restrictions, which characterizes wheel–workpiece interactions through analyzing high-frequency variations in structural impedance [18]. Junior et al. [19] optimized wavelet-assisted piezoelectric EMI monitoring for grinding wheel condition evaluation, yet this method is only applicable during wheel dressing and fails to achieve synchronous monitoring of both thermal damage and abrasive wear.

Spindle power monitoring acts as a low-cost auxiliary sensing scheme, where spindle driving power is utilized to reflect instantaneous grinding loads [20,21]. Operators can adjust feed speed and grinding depth according to real-time power fluctuations. Even so, spindle power only reflects the overall energy consumption of the grinding system and lacks characteristic parameters to quantify grinding burn severity or wheel wear extent, resulting in low sensitivity for early warning of quality defects.

Electrostatic monitoring technology was initially developed to detect charged abrasive debris within gas flow channels of aero-engines and gas turbines [22]. Its fundamental principle lies in the capability of electrostatic sensors to capture charged tribological wear particles [23]. When electrostatic sensors are installed at engine nozzles to collect electrostatic signals from wear debris, signal analysis enables the identification of equipment faults including blade adhesive friction, combustion chamber corrosion, deteriorated combustion performance and coating abrasion. Electrostatic monitoring has also found practical applications in rolling bearing fault diagnosis [24,25], which realizes early warning of potential bearing failures by detecting electrostatic charges carried by wear particles. Benefiting from its non-contact layout, ultra-high sensitivity and convenient hardware installation, electrostatic sensing has evolved into a key supporting technology for fault diagnosis and health management of rotating machinery [26,27]. Multi-source data fusion combining electrostatic signals, vibration signals and native equipment operating parameters can construct comprehensive equipment health evaluation models to deliver reliable decision support for predictive maintenance.

Grinding processes involve rubbing, plowing and cutting actions [28]. Rubbing and plowing are dominated by frictional effects, whereas cutting combines frictional interaction and material fracture. Both interfacial friction and material fracture can induce electrostatic charging of solid surfaces [29]. Intensive frictional contact between the workpiece and grinding wheel during grinding inevitably triggers charge transfer, causing electrostatic charging of both contact components. Separated chips and fractured abrasive grits under friction also carry electrostatic charges, which establishes the physical basis for electrostatic monitoring. Previous investigations have verified the positive correlation between wheel wear evolution and electrostatic responses [30]. Nevertheless, research concerning the relationship between wheel wear and electrostatic signals remains limited. Few studies have analyzed the influence of such signals on machined surface topography, and the correlation between grinding parameters and electrostatic signals is not well understood. Moreover, systematic quantitative analyses covering the entire grinding cycle are still insufficient.

Against this research background and built upon our previous work, this paper further advances the research on electrostatic induction-based online monitoring of grinding processes and deeply analyzes the electrostatic charging mechanism during grinding. Grinding experiments are carried out to explore the correlation laws between grinding parameters and electrostatic signals. On the basis of experimental results, the mapping relationship between electrostatic characteristics and machining parameters is derived, and online monitoring of grinding parameters is realized using electrostatic signals. Furthermore, comparative analyses with the conventional grinding force monitoring method are conducted. Finally, effective augmentation of monitoring data is implemented to achieve online identification of grinding wheel wear states.

## 2. Electrostatic Monitoring Process

**Figure 1:**
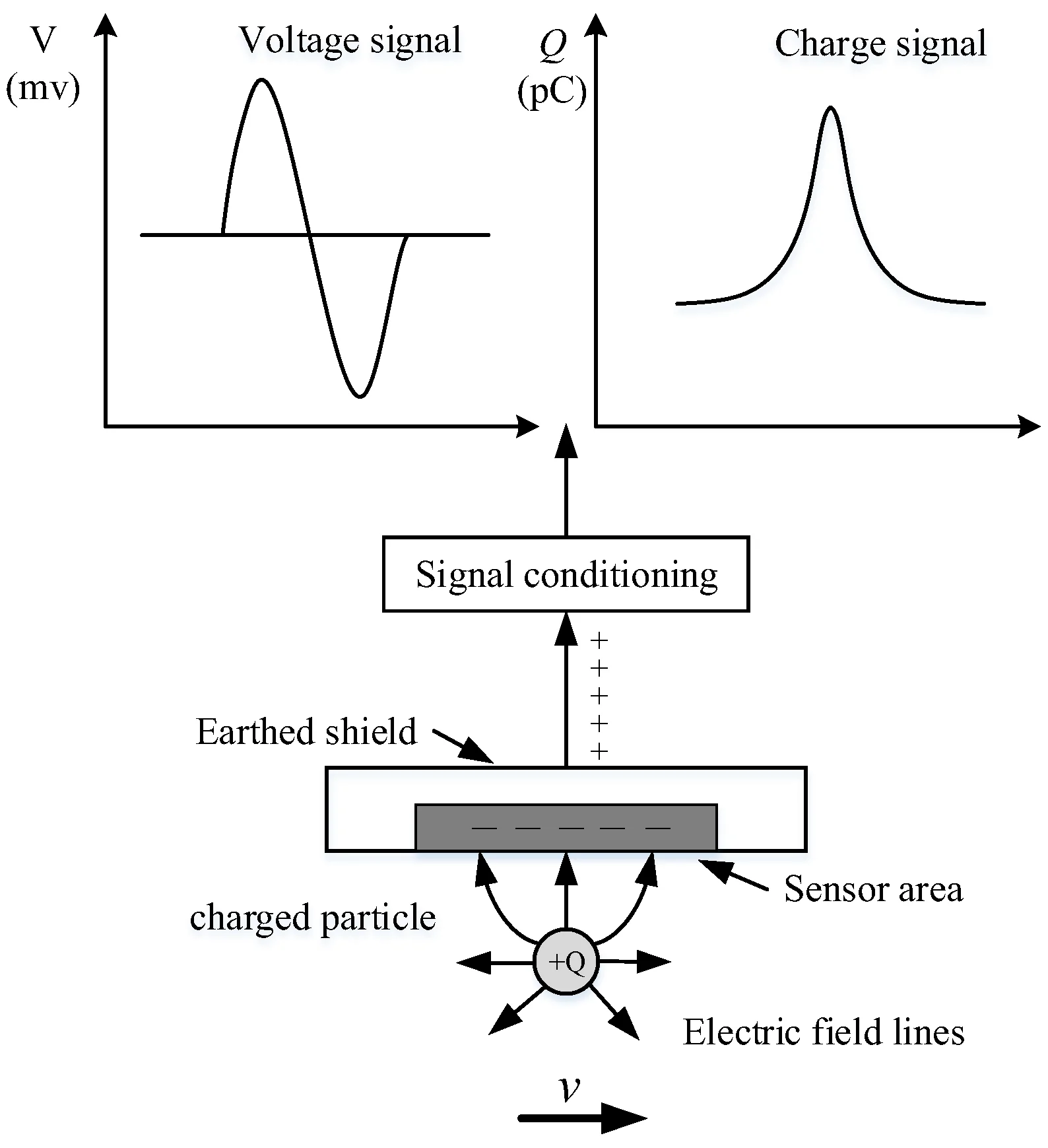
Principle of electrostatic monitoring.

As illustrated in Figure 1, the working principle of the electrostatic sensor is described as follows: When charged particles pass through the sensing area, the external electric field induces the redistribution of free electrons within the metal probe, thereby generating surface induced charges with opposite polarity to the moving particles. Such time-varying charge redistribution produces a transient induced current, which further yields an induced electromotive force in the measurement circuit.

### 2.1. Electrostatic Sensor Model

**Figure 2:**
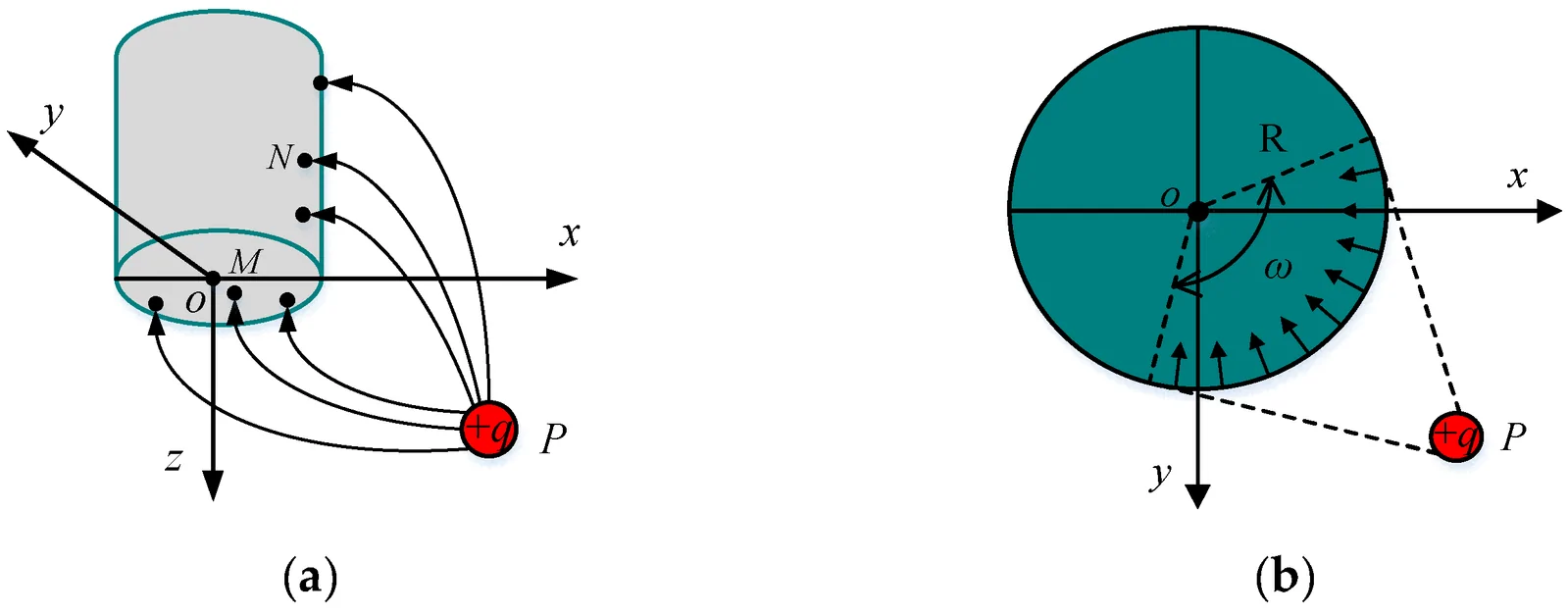
Electric field line distribution of the sensor: (**a**) model of the electric field lines; (**b**) effective monitoring region of the sensor.

As shown in Figure 2 (adapted from [30]), when a point charge +q passes by the rod-shaped electrostatic sensor, part of the electric field lines emanating from +q terminates perpendicularly on the bottom and side surfaces of the probe, inducing equivalent surface charges over the sensor. For quantitative modeling, a Cartesian coordinate system is established with its origin *O* located at the center of the probe’s lower end face. The instantaneous position of the moving point charge is defined as *P*(x, y, z). Correspondingly, *M*(u, v, w) represents an arbitrary point on the bottom surface, and *N*(i, j, k) denotes an arbitrary point on the lateral surface. The induced charge distributions on the two surfaces are modeled and analyzed independently in this study.

The amount of charge *Q*_1_ induced on the entire end face is [30]:

(1) Q1=qz4π∫02πdθ∫0Rr(x−rcosθ)2+(y−rsinθ)2+z232dr,

where *q*: the charge magnitude; *R*: the radius of the probe end face; *r*: the distance from point M to the circle center; *θ*: the angle between oM and the *x*-axis.

The total induced charge on the side of the probe is

(2) Q2=q4π∬S2(i−si2x−sijy)2+(j−sijx−sj2y)212(x−i)2+(y−j)2+(z−k)232dS2,

where *S*_2_: the effective area of the side of the probe.

Let the effective induced length of the probe be *L*. By calculating the angle *ω* from the coordinates of point P, Equation (3) is obtained.

(3) $$Q_{2}=\frac{q}{4\pi }\int_{\arctan\left|\frac{y}{x}\right|-\arccos\left|\frac{R}{\sqrt{x^{2}+y^{2}}}\right|}^{\arctan\left|\frac{y}{x}\right|+\arccos\left|\frac{R}{\sqrt{x^{2}+y^{2}}}\right|}d\omega \int_{-L}^{0}\frac{\left|R-\left|x\right|\cos\omega -\left|y\right|\sin\omega \right|}{{\left[{\left(\left|x\right|-R\cos\omega \right)}^{2}+{\left(\left|y\right|-R\sin\omega \right)}^{2}+{\left(z-k\right)}^{2}\right]}^{3/2}}Rdk,$$

where *L*: the probe length.

The total induced charge on the probe, *Q*, is given by

(4) Q=qz4π∫02πdθ∫0Rr(x−rcosθ)2+(y−rsinθ)2+z232dr+q4π∫arctanyx−arccosRx2+y2arctanyx+arccosRx2+y2dω∫−L0R−xcosω−ysinω(x−Rcosω)2+(y−Rsinω)2+(z−k)23/2Rdk,

Equation (4) shows that the induced charge on the probe increases linearly with the magnitude of the measured charge and the effective sensing area, whereas it decreases with the measurement distance.

### 2.2. Charging Mechanism in Grinding

#### 2.2.1. Analysis of Grinding Wheel Charging

**Figure 3:**
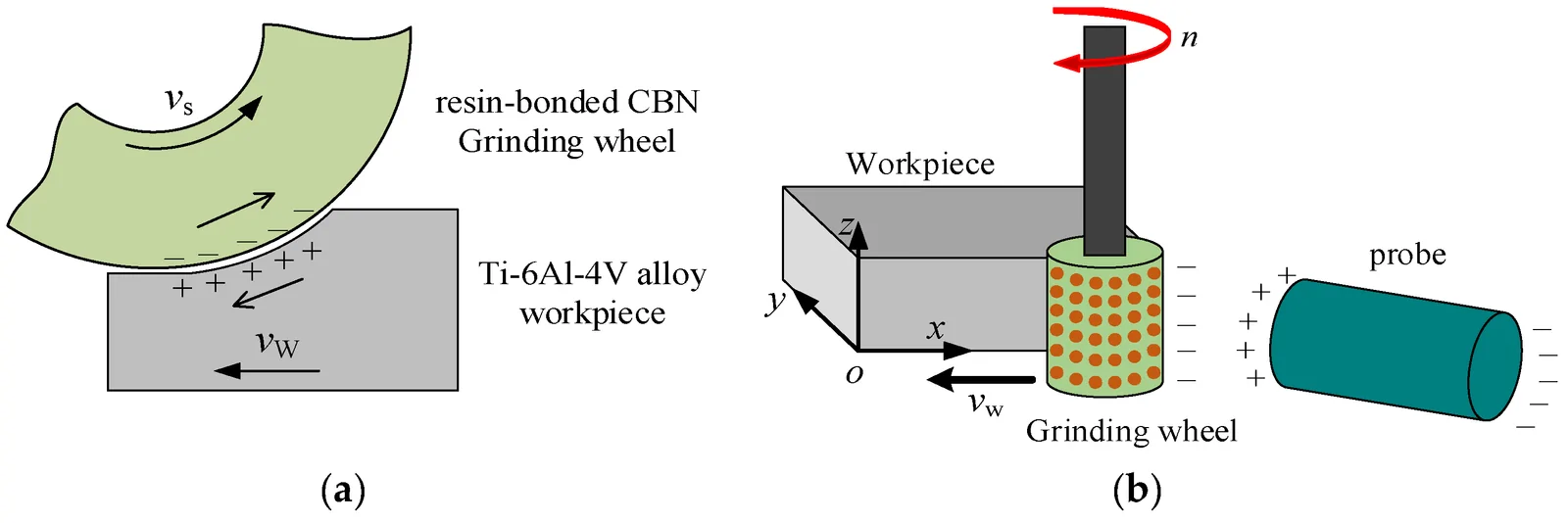
Grinding process scheme: (**a**) charge transfer of the grinding wheel; (**b**) probe induction.

Intense frictional interaction takes place at the contact interface between the grinding wheel and workpiece during grinding, which initiates triboelectric charge transfer and leads to electrostatic charging of both contact components. As shown in Figure 3a, electron migration occurs from the titanium alloy toward cubic boron nitride (CBN) abrasives and the entire resin-bonded grinding wheel upon grinding contact. Titanium alloy possesses a lower work function and tends to lose electrons readily, whereas CBN abrasives and the wheel binder matrix feature high electron affinity and strong electron-trapping capacity. As depicted in Figure 3b (adapted from [30]), when the electrostatic probe is positioned adjacent to the grinding wheel, the external electric field induces redistribution of free electrons within the probe. Accordingly, the sensor can acquire the induced electrostatic signal originating from the charged grinding wheel.

#### 2.2.2. Charge Analysis of Chips and Abrasive Grains

**Figure 4:**
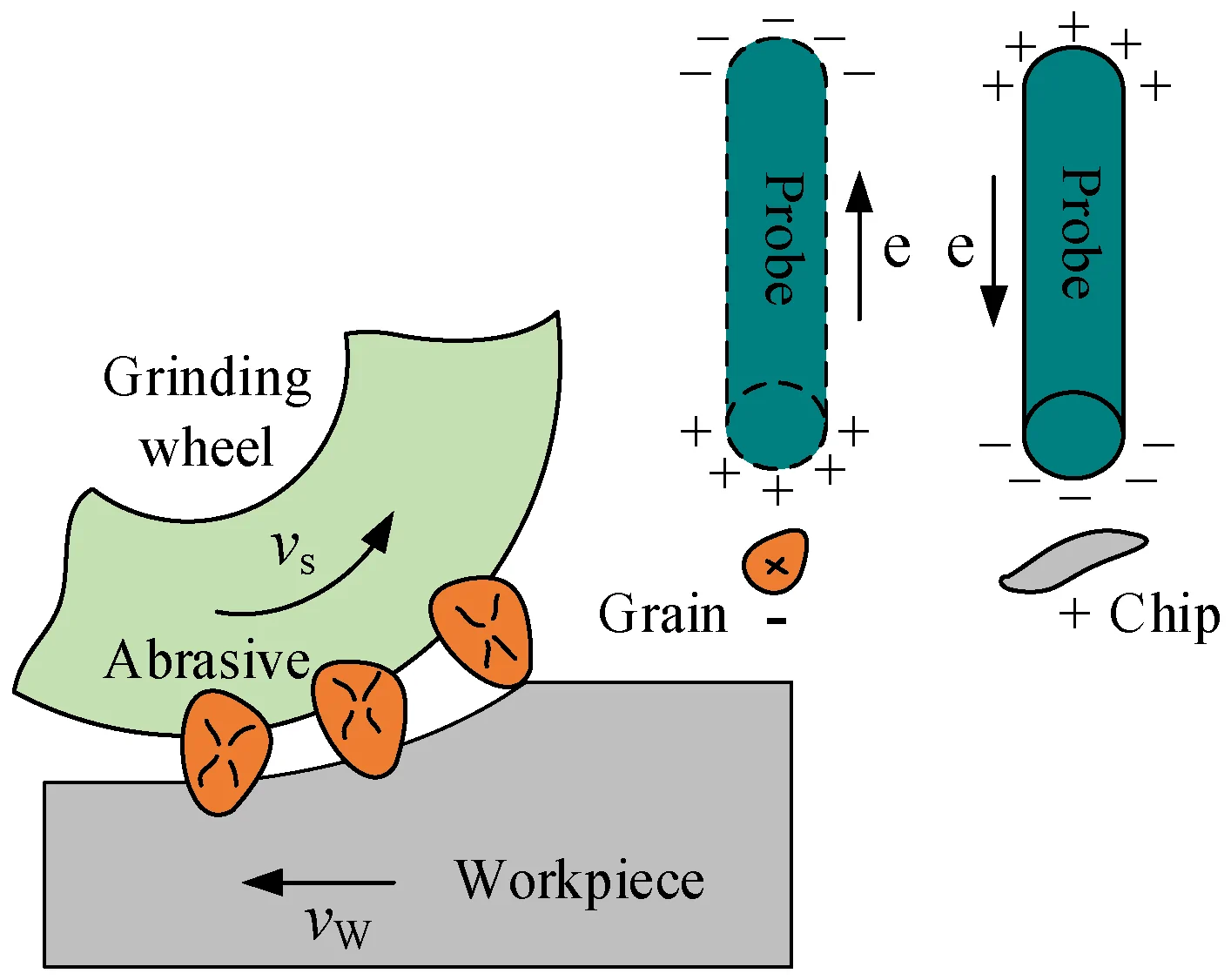
Measurement mechanism of abrasive grains and chips.

Once abrasive grains detach from the grinding wheel surface, the resulting isolated particles lose their conductive pathways. Electrons transferred during frictional interactions are confined within the grains, rendering the detached abrasives negatively charged. Similarly, grinding chips stripped from the titanium alloy workpiece lack conductive pathways and thus carry positive charges. In theory, the type of charged particles can be distinguished based on their charge polarity. Nevertheless, environmental conditions and foreign-particle interference introduce inherent identification uncertainty. Relevant information can still be extracted by comprehensively evaluating electrostatic signal features. As illustrated in Figure 4, when these charged chips or abrasive grains travel past the sensor probe, they induce redistribution of free electrons within the probe. Combined with subsequent signal conditioning and analysis, the corresponding electrostatic signature can be obtained.

## 3. Experiment

**Figure 5:**
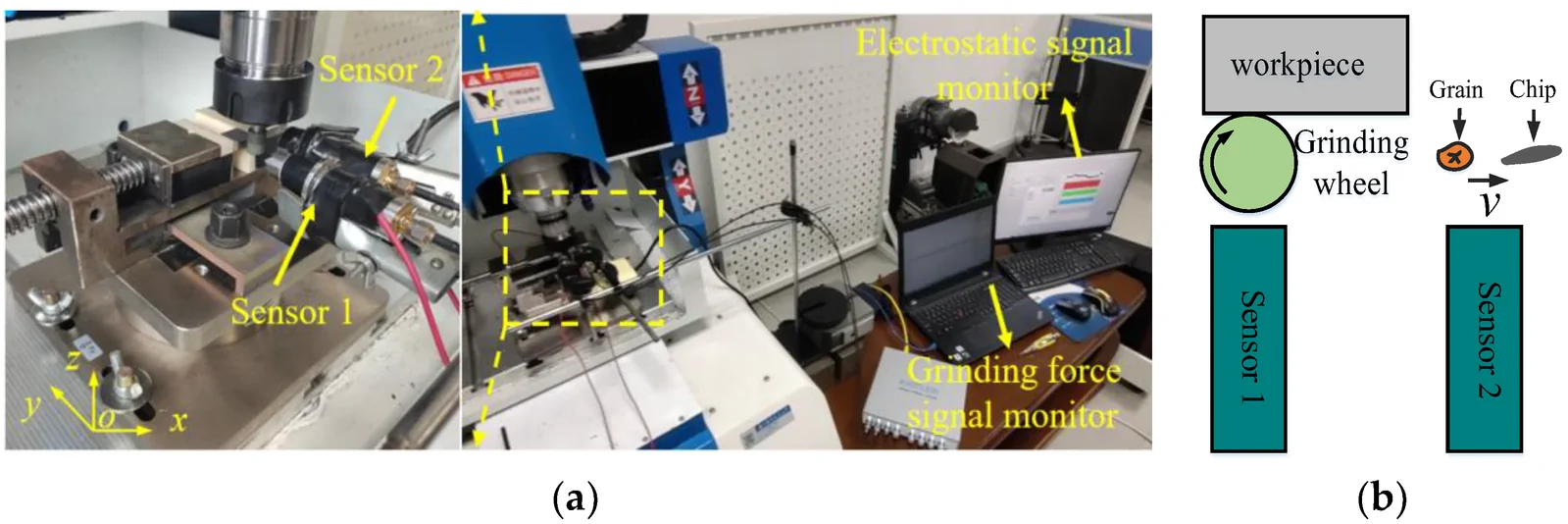
Electrostatic monitoring system: (**a**) experimental setup; (**b**) monitoring principle.

As shown in Figure 5a, the experimental test bench was constructed based on a three-axis CNC engraving machine, on which the grinding wheel was used to machine the lateral surface of the workpiece. A triaxial dynamometer mounted on the worktable acquired grinding force data in real time. The z-direction forces generated by individual abrasive grains counteract one another, leading to a negligible net load along the *z*-axis; therefore, only forces in the x and y directions are analyzed in this study.

Surface charge variation occurs immediately upon contact between the grinding wheel and workpiece. Accordingly, Sensor 1 was deployed to monitor such electrostatic variations. Grinding generates abundant grinding chips and detaches abrasive grains, and Sensor 2 was arranged to capture the electrostatic signals originating from these particles. The relative positional layout of Sensor 1 and Sensor 2 with respect to the grinding wheel and workpiece is presented in Figure 5b.

Two rod-shaped electrostatic sensors were deployed throughout the experiments. Each sensor features an overall length of 90 mm, alongside a metal shielding housing with an outer diameter of 12 mm and a probe tip diameter of 9 mm. The probe of Sensor 1 was oriented perpendicularly towards the grinding surface of the workpiece, maintaining a stand-off distance of 20 mm. Sensor 2 was arranged parallel to Sensor 1 at an identical height on its right-hand side. The axial center-to-center spacing between the two sensors was 15 mm, and Sensor 2 was positioned with a stand-off distance of 10 mm away from the workpiece grinding surface.

Metal shielding housings were adopted to mitigate external electromagnetic interference, and all shielding shells were reliably grounded to isolate stray noise interfering with the sensor probes. The charge amplifier was set to a fixed gain of 10 mV/pC. The background noise level reached ±4 pC for Sensor 1 and ±0.1 pC for Sensor 2. Electrostatic signals were sampled at 25 kHz, and a low-pass filter with a cutoff frequency of 20 Hz was implemented for signal denoising. All grinding experiments were carried out under a steady ambient relative humidity of 45 ± 5% RH.

The electrostatic sensors were calibrated via an oil charging calibration approach. A favorable linear correlation was identified between sensor pulse outputs and the charge magnitude of charged oil droplets, validating the measurement accuracy and stability of the developed sensors.

A six-component dynamometer (ADPW-6D-500, Aipike Electronic Technology Co., Ltd., Bengbu, China) was used to capture grinding force signals at a sampling frequency of 1500 Hz. A low-pass filter with a cutoff frequency of 50 Hz was applied to attenuate high-frequency noise originating from spindle vibration and power frequency electromagnetic disturbance. The electrostatic acquisition system and force measurement system operated independently during testing, eliminating mutual interference.

The workpieces were made of Ti-6Al-4V titanium alloy, with dimensions of 15 mm × 15 mm × 5 mm. Table 1 and Table 2 summarize the nominal chemical composition and key thermophysical properties of this alloy, respectively.

Resin-bonded cubic boron nitride (CBN) grinding wheels feature favorable self-sharpening performance, low grinding forces, low grinding temperatures, and the capacity to produce superior machined surface quality. Furthermore, CBN exhibits chemical inertness toward iron-group elements, rendering it well-suited for titanium alloy grinding. In this study, a straight CBN grinding wheel with an abrasive grain size ranging from 125 μm to 150 μm and a grain concentration of 100 was adopted.

## 4. Results and Discussion

**Figure 6:**
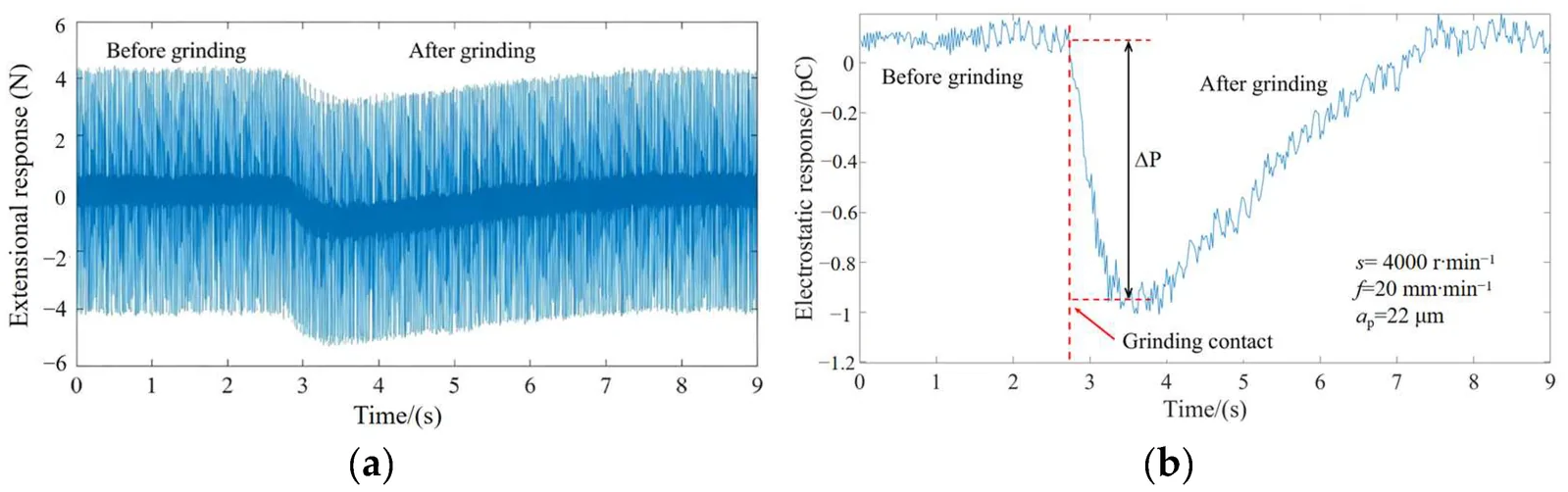
Electrostatic signal of grinding wheel: (**a**) initial signal; (**b**) simplified signal.

As analyzed in Section 2.2, both the grinding wheel and workpiece generate electrostatic charges once contact occurs. Electrostatic Sensor 1 captures the charging state of the grinding wheel, and an abrupt signal jump can be observed the moment contact is established. The variation trend illustrated in Figure 6a agrees well with the preceding theoretical analysis. Nevertheless, the signal trend is indistinct owing to power line interference. To visualize this trend more intuitively, low-pass filtering is performed, and raw signals are processed by averaging within a 0.02 s sliding time window. After signal processing, the noise level of the electrostatic sensors is significantly reduced to within ±0.1 pC, which facilitates signal monitoring. The processed variation curve is presented in Figure 6b.

**Figure 7:**
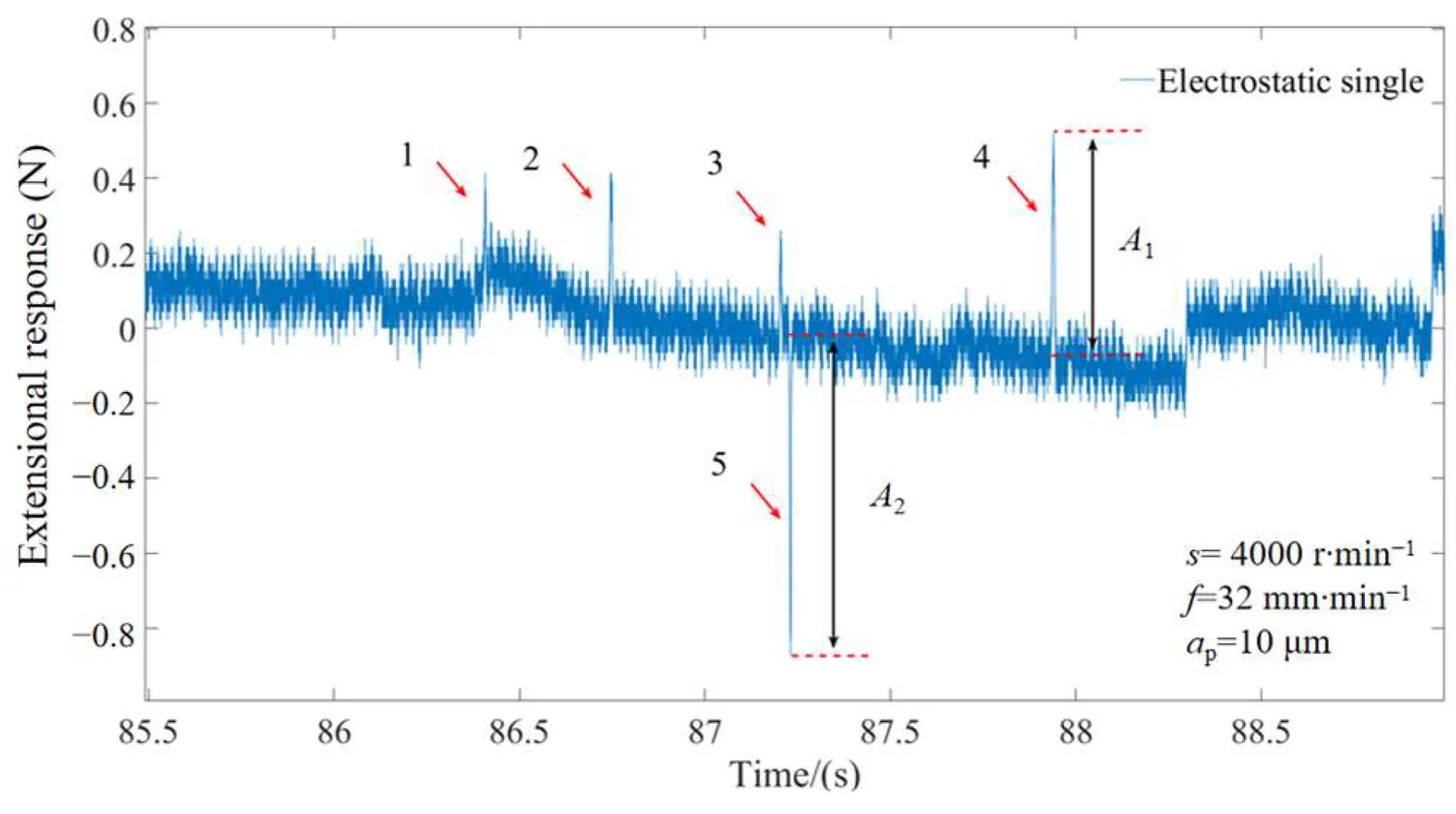
Electrostatic signals of chips and abrasive grains.

The signal captured by Electrostatic Sensor 2 during grinding is illustrated in Figure 7. The waveform consists of pulses generated by both grinding chips and detached abrasive grains, whose amplitudes exhibit obvious non-uniformity. A primary cause lies in the continuously changing minimum stand-off distance between particles and the sensor. Since the induced peak magnitude is inversely proportional to this distance, individual chips or abrasive grains produce pulses of varying heights.

Single-factor grinding experiments were performed to investigate the individual effects of wheel speed, depth of cut, and feed rate on the electrostatic signal.

### 4.1. Influence of Grinding Wheel Speed on the Electrostatic Signal

The machining parameters used at each rotational speed are summarized in Table 3.

**Figure 8:**
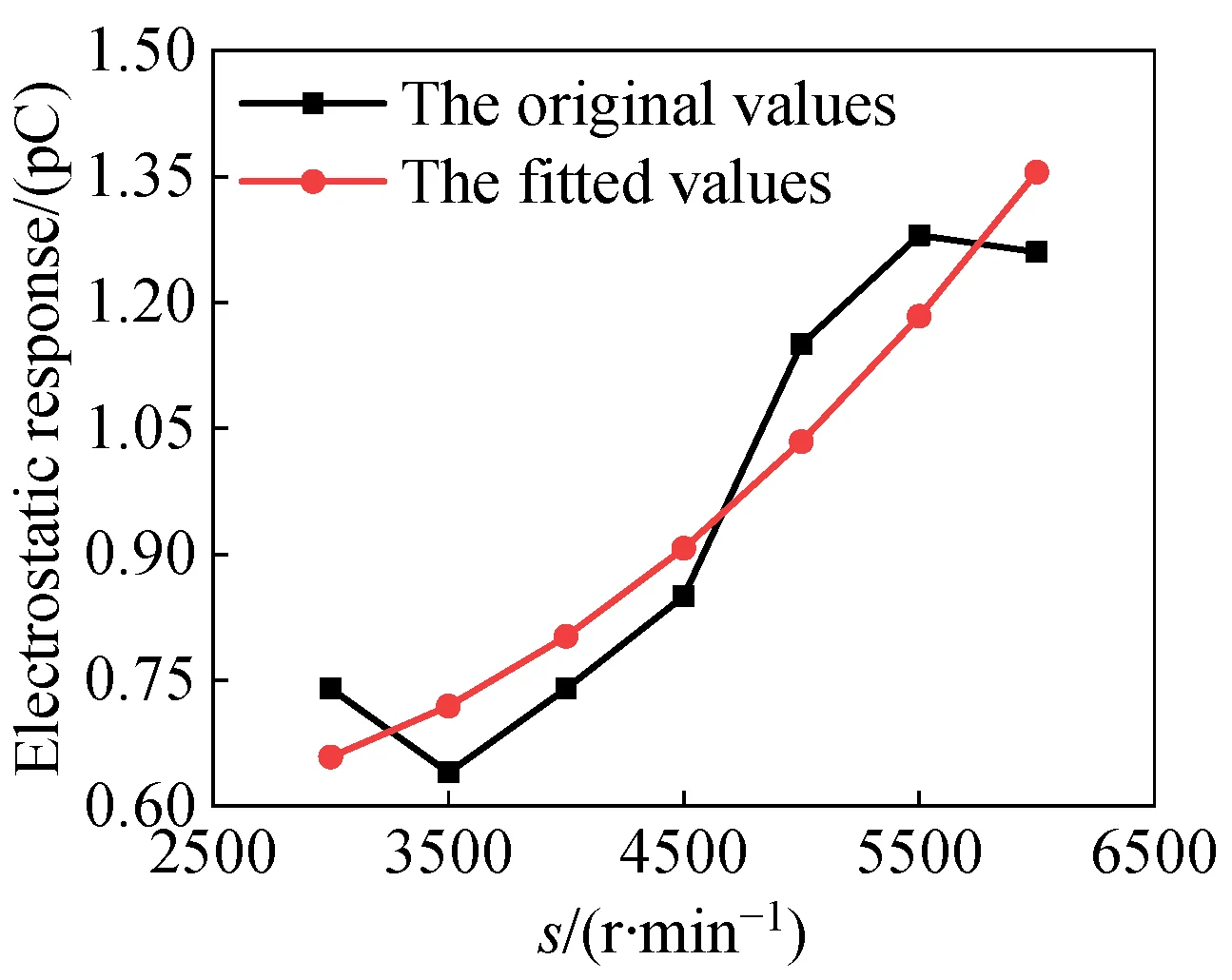
Variation in the charge of the grinding wheel at different speeds.

**Figure 9:**
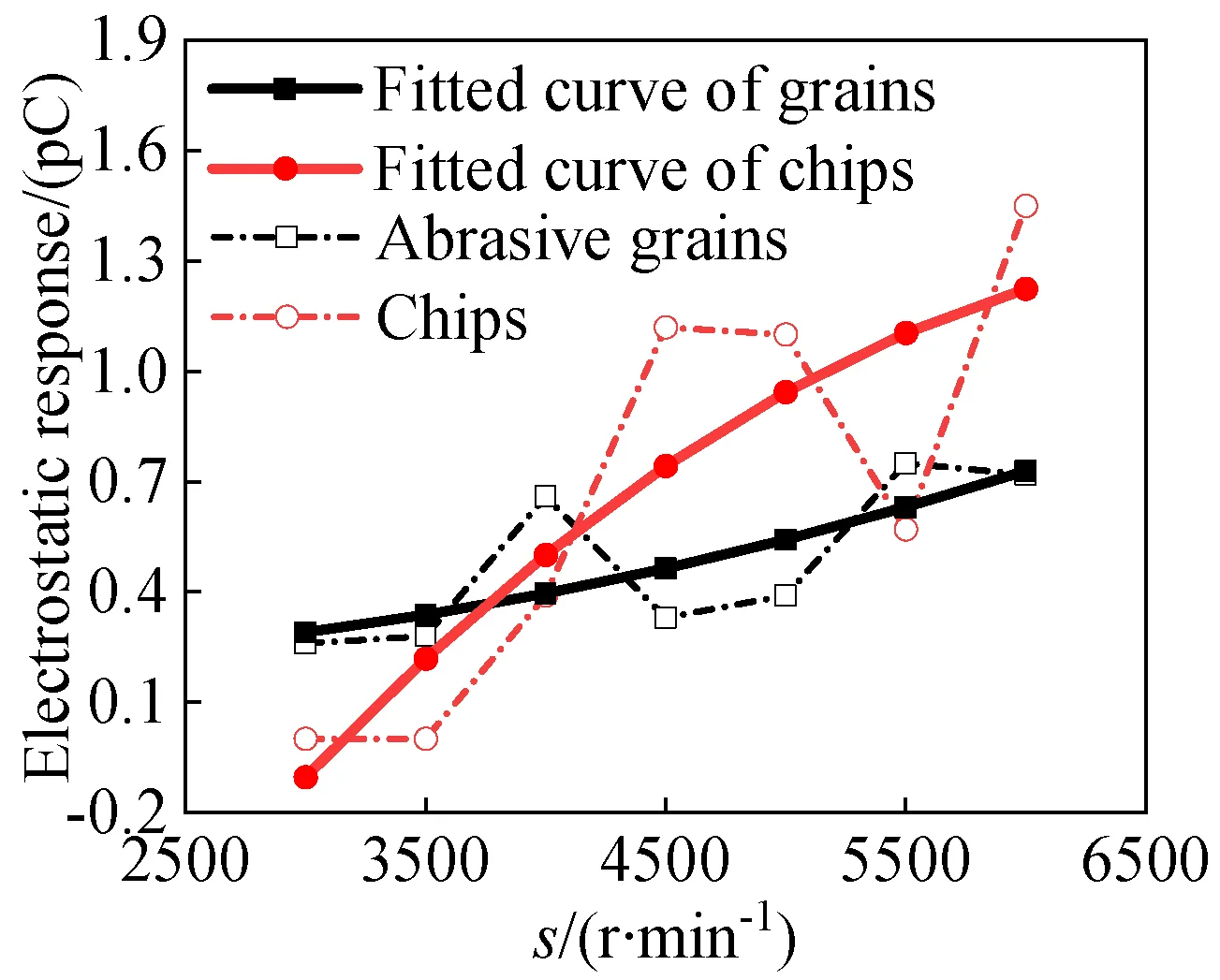
Peak-induced pulse amplitudes from chips and dislodged abrasive grains at different grinding wheel speeds.

Triboelectric charging is triggered by interfacial friction at the instant of wheel–workpiece contact. Figure 8 shows the total accumulated charge on the grinding wheel under different rotational speeds, while Figure 9 presents the corresponding variations in the maximum induced charge produced by grinding chips and detached abrasive grains.

As shown in Figure 8, the accumulated charge on the grinding wheel increases with rotational speed. A higher wheel speed strengthens the frictional interaction between the grinding wheel and workpiece, generating more charge accumulated on the wheel surface. When the rotational speed rises from 3000 r·min^−1^ to 6000 r·min^−1^, the charge magnitude grows by 106%. The experimental results agree well with the foregoing theoretical analysis, and all repeated tests follow consistent variation tendencies. This finding confirms that the charge level of the grinding wheel can serve as a characteristic monitoring parameter for reflecting wheel rotational speed.

Figure 9 reveals that the peak-induced charge originating from grinding chips and detached abrasive grains fluctuates as the grinding speed rises, while exhibiting an overall upward tendency. This phenomenon can be interpreted as follows: A higher rotational speed of the grinding wheel intensifies frictional interaction, and the elevated frictional work transfers more electrostatic charge to grinding chips and detached grains, thereby producing induced pulse signals with larger amplitudes. Such signal fluctuations stem from the random variation in the minimum distance between the probe center and moving chips as well as detached abrasive grains, yet they do not mask the overall variation trend.

**Figure 10:**
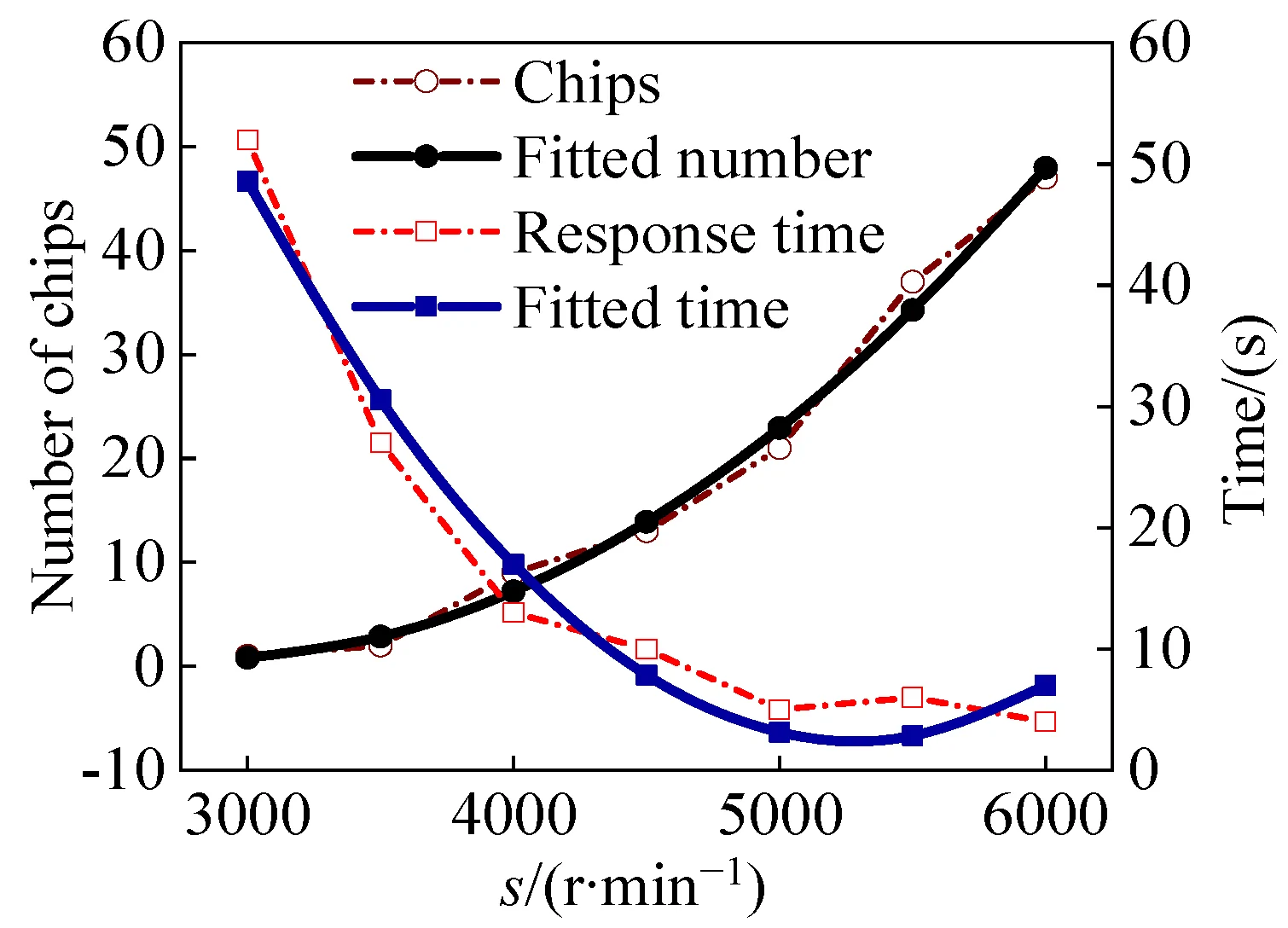
Chip pulse count and time to first chip pulse versus grinding wheel speed.

Figure 10 illustrates the total number of chip-induced pulses captured throughout grinding, as well as the time interval between wheel–workpiece contact and the first detectable chip-induced pulse under different wheel speeds. Both metrics reveal that wheel speed exhibits a positive correlation with pulse count and a negative correlation with the delay of the first detected pulse. Such behavior originates from three concurrent effects: (1) at higher rotational speeds, the ejection velocity of certain grinding chips becomes sufficiently high to transport these particles into the probe’s sensing zone; (2) the overall chip generation rate increases with wheel speed, allowing more particles to travel through the effective monitoring region; (3) enhanced triboelectric charging of the grinding wheel and intensified wheel–workpiece friction elevate the charge magnitude carried by individual chips, thereby increasing the probability of successful detection.

**Figure 11:**
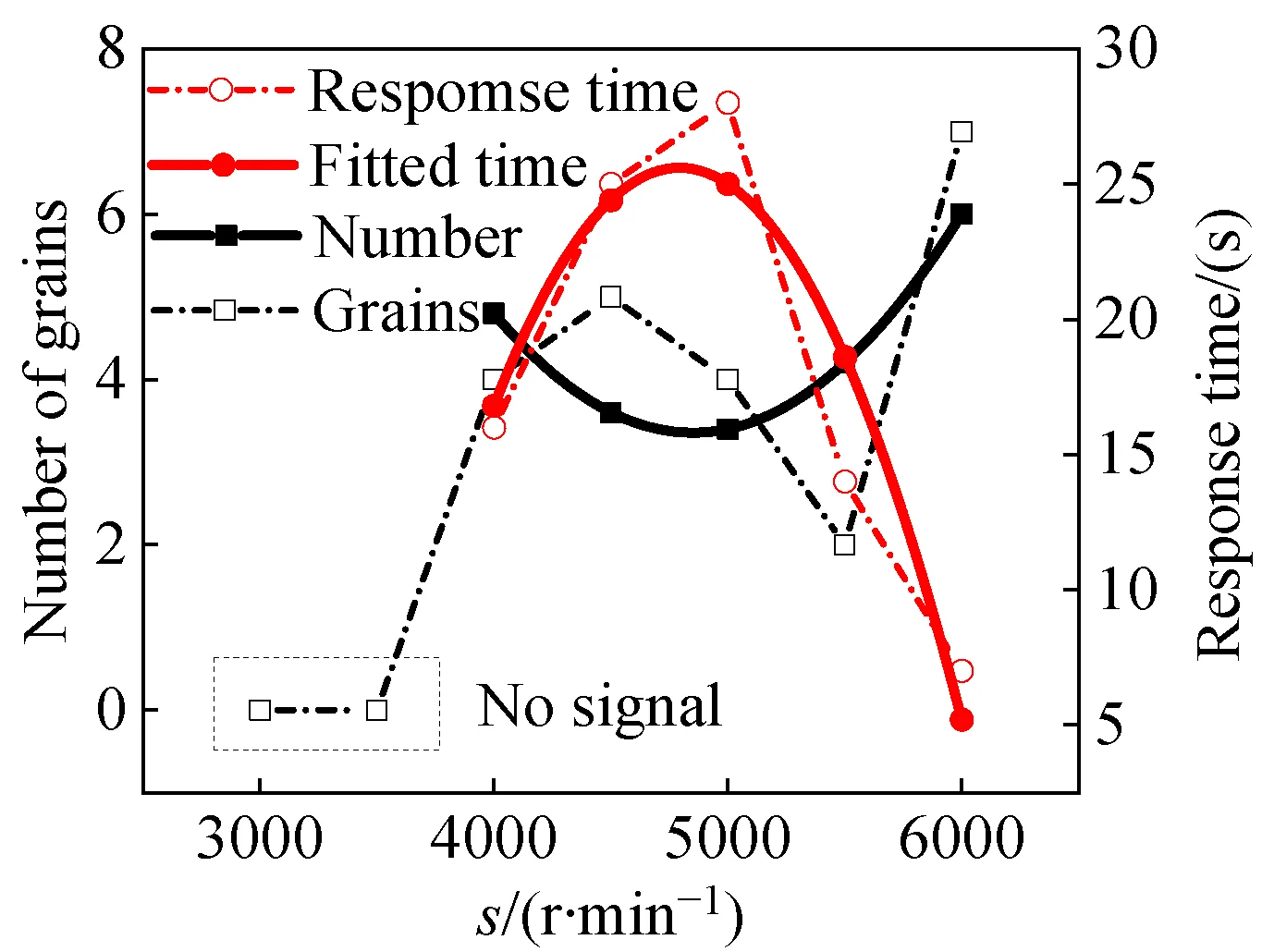
Dislodged-grain pulse count and time to first grain pulse versus grinding wheel speed.

Figure 11 summarizes the number of pulses originating from detached abrasive grains and the time interval from grinding initiation to the first detected grain-induced pulse under various wheel speeds. Since abrasive grain detachment is an inherently stochastic process, neither the pulse count nor the arrival time of the first pulse shows an evident correlation with wheel speed. Accordingly, these two metrics offer limited capability for wheel rotational speed estimation.

### 4.2. Influence of Depth of Cut on the Electrostatic Signal

The machining parameters under different depths of cut are listed in Table 4.

**Figure 12:**
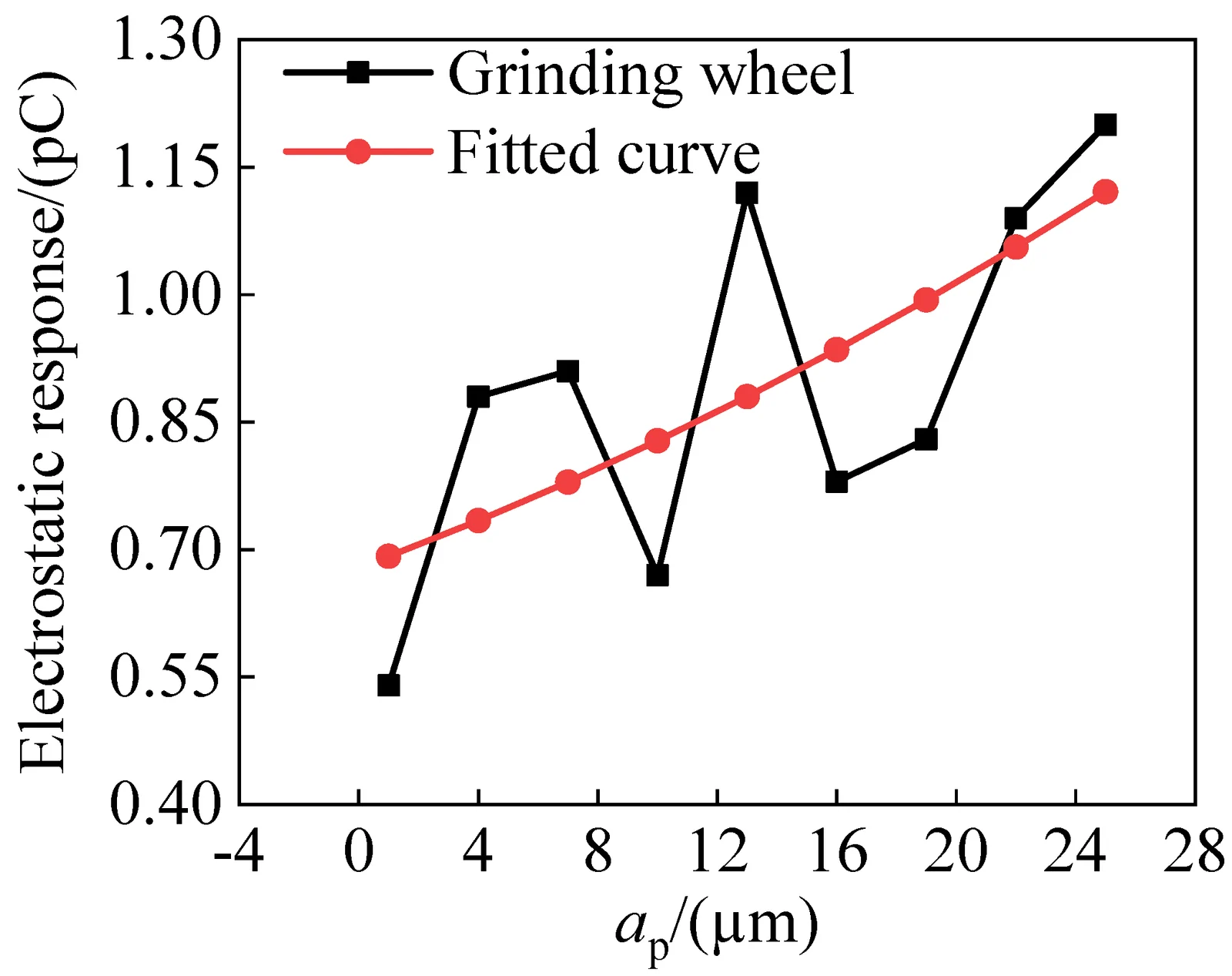
Variation in wheel charge versus depth of cut.

As illustrated in Figure 12, notable local fluctuations exist within the measured data. Nevertheless, a fundamental physical tendency is that a larger depth of cut induces stronger frictional interaction between the grinding wheel and workpiece. Accordingly, the accumulated charge on the grinding wheel increases as the depth of cut rises. Fitting the experimental data using a second-order polynomial function attenuates random discrete noise and clearly reveals the underlying variation trend. As the depth of cut increases from 1 μm to 25 μm, the induced charge magnitude grows by 62%. The above experimental results confirm that the charge level of the grinding wheel can serve as a monitoring indicator for tracking variations in depth of cut.

**Figure 13:**
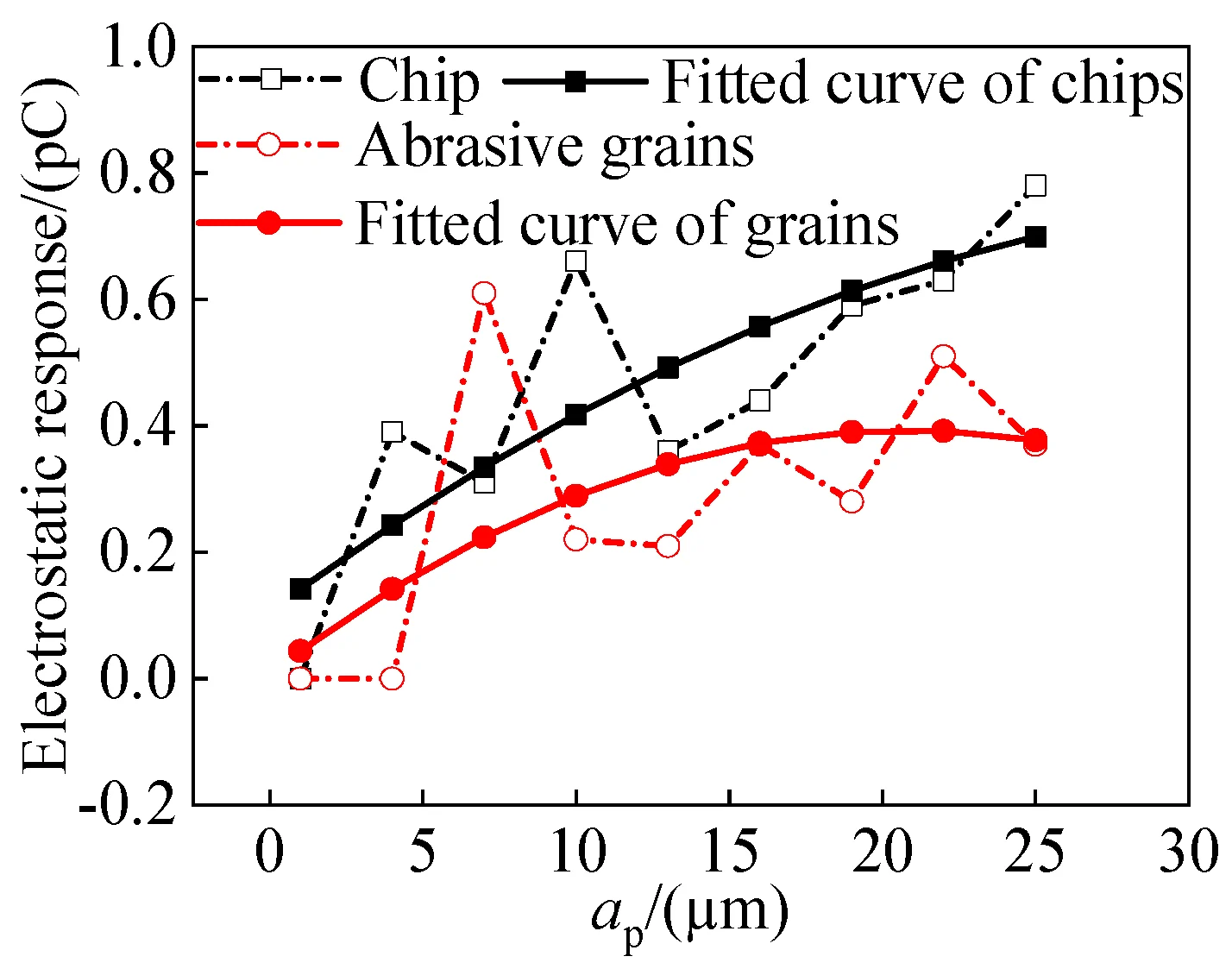
Peak-induced pulse amplitudes from chips and dislodged abrasive grains versus grinding depth.

As shown in Figure 13, despite random variations in the minimum measuring distance, the peak charge carried by grinding chips and detached abrasive grains generally increases with the rise in depth of cut. Second-order polynomial fitting of the electrostatic measurement data mitigates the impact of random errors and reveals an approximately linear monotonic relationship between the maximum particle charge and depth of cut. This trend can be interpreted as follows: A larger depth of cut enlarges the contact area between the grinding wheel and workpiece and intensifies frictional interaction. Both effects promote triboelectric charge transfer, thereby elevating the total charge carried by grinding chips and detached abrasive grains.

**Figure 14:**
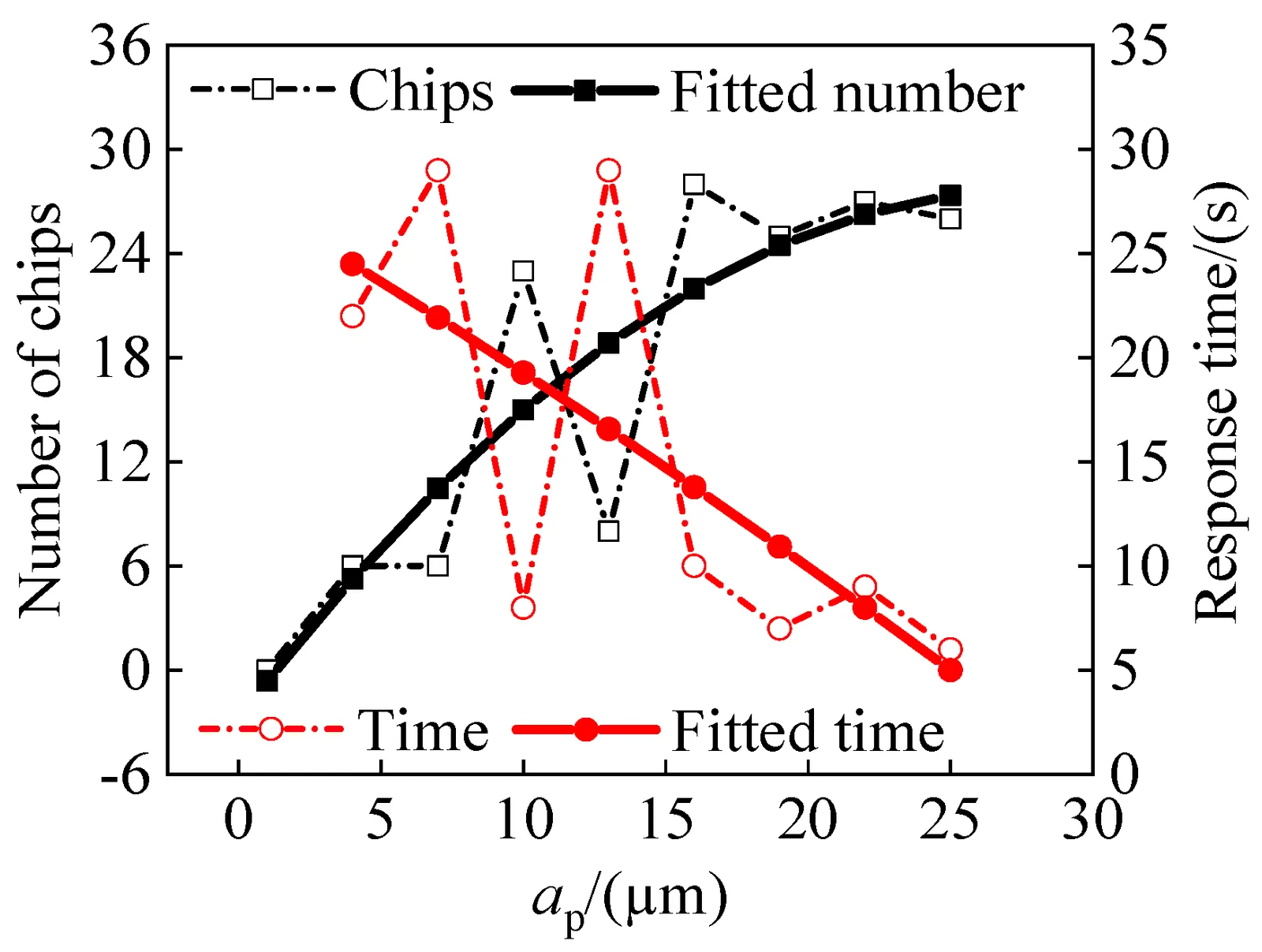
Chip pulse count and time to first chip pulse versus depth of cut.

Figure 14 indicates that both the count of chip-induced pulses and the arrival time of the first chip pulse exhibit initial fluctuations as the depth of cut increases. Nevertheless, a distinct trend is observed after polynomial fitting: the number of chip pulses grows with rising depth of cut, while the delay of the first pulse decreases. A larger depth of cut strengthens frictional interaction and elevates the charge magnitude of individual grinding chips. Consequently, particles formerly falling below the detection threshold become detectable, leading to an increased total pulse count. The elevated charge level also enables earlier detection of particles and shortens the delay of the first pulse. These experimental findings confirm that the chip pulse count and the first-pulse delay can serve as reliable indicators for evaluating the depth of cut.

**Figure 15:**
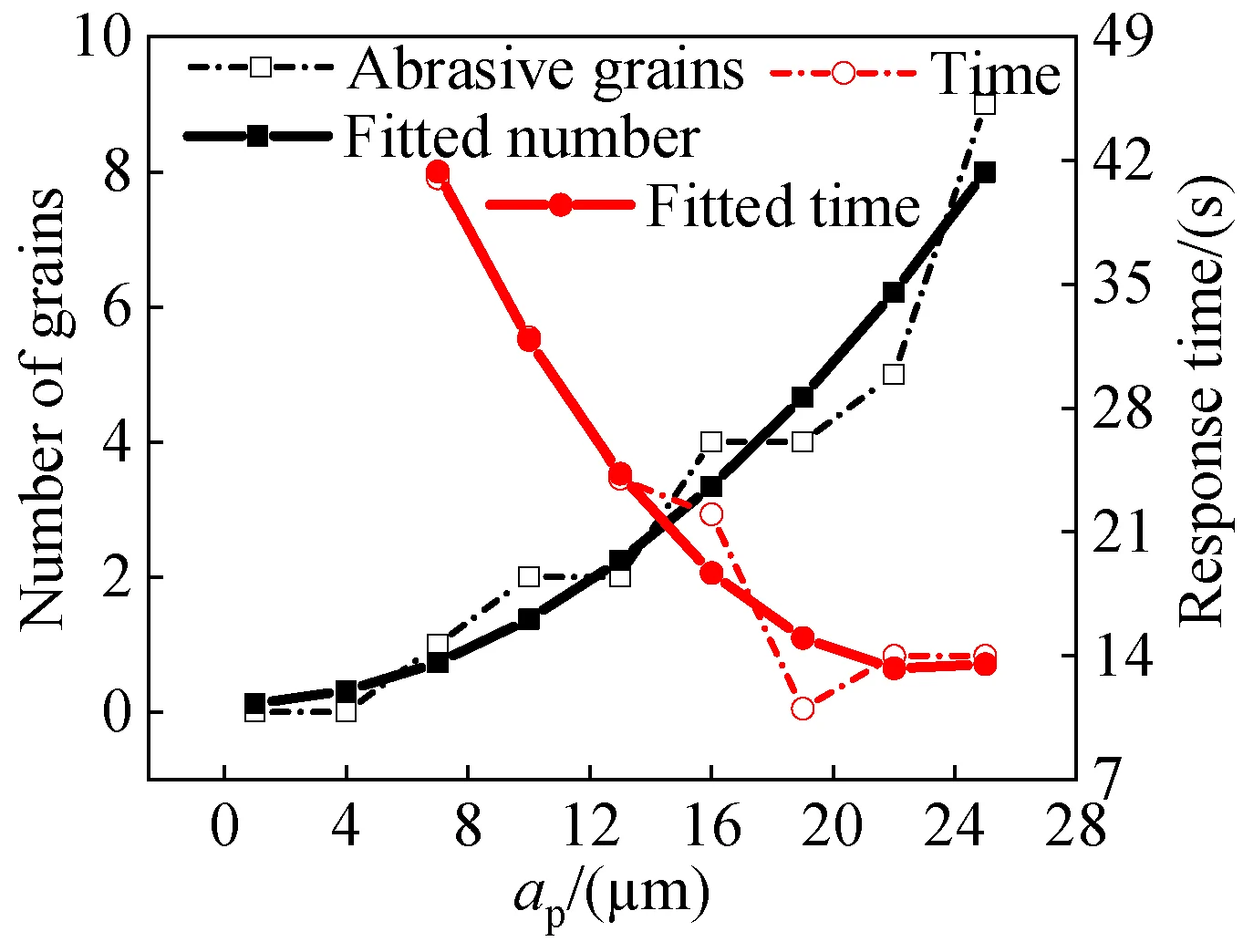
Dislodged-grain pulse count and time to first grain pulse versus grinding depth.

Figure 15 reveals that as the depth of cut increases, pulses from detached abrasive grains grow in number overall, while the arrival time of the first grain-induced pulse declines. A larger depth of cut strengthens wheel–workpiece frictional interaction, raising the charge on individual abrasives and allowing more pulses to exceed the detection threshold. Meanwhile, higher mechanical load facilitates grain detachment and increases the quantity of charged grains traveling past the probe. Collectively, these factors indicate that the pulse count and reduced first-pulse delay can serve as valid indicators of depth of cut.

### 4.3. Influence of Grinding Feed Rate on the Electrostatic Signal

The experimental parameters corresponding to different feed rates are listed in Table 5.

**Figure 16:**
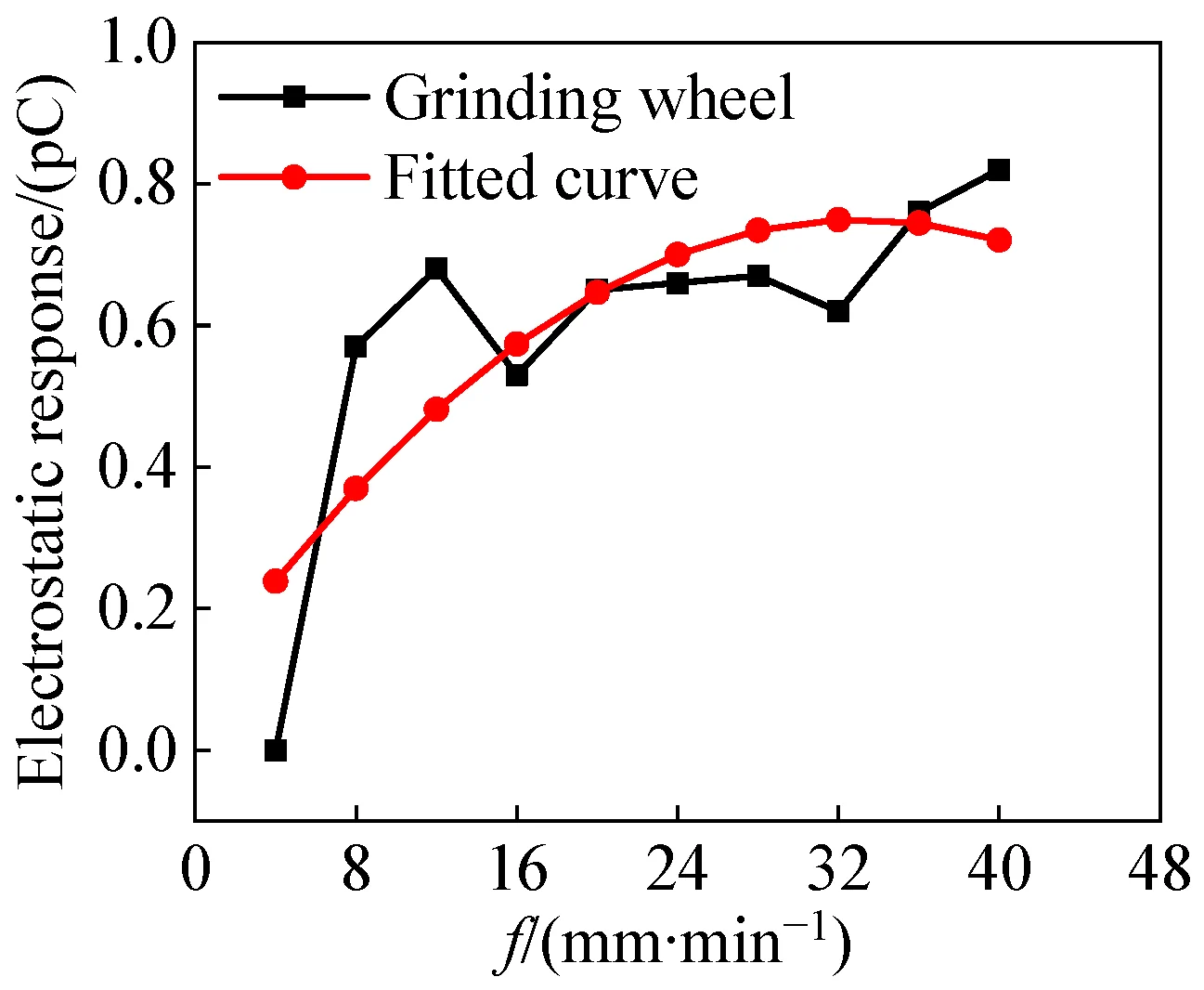
Wheel charge versus feed rate.

As shown in Figure 16, the accumulated electrostatic charge on the grinding wheel rises monotonically with feed rate. As the feed rate increases from 4 mm·min^−1^ to 40 mm·min^−1^, the charge signal rises by 202.5%. This finding confirms that the electrostatic characteristic of the grinding wheel serves as a reliable indicator for tracking feed rate variations.

**Figure 17:**
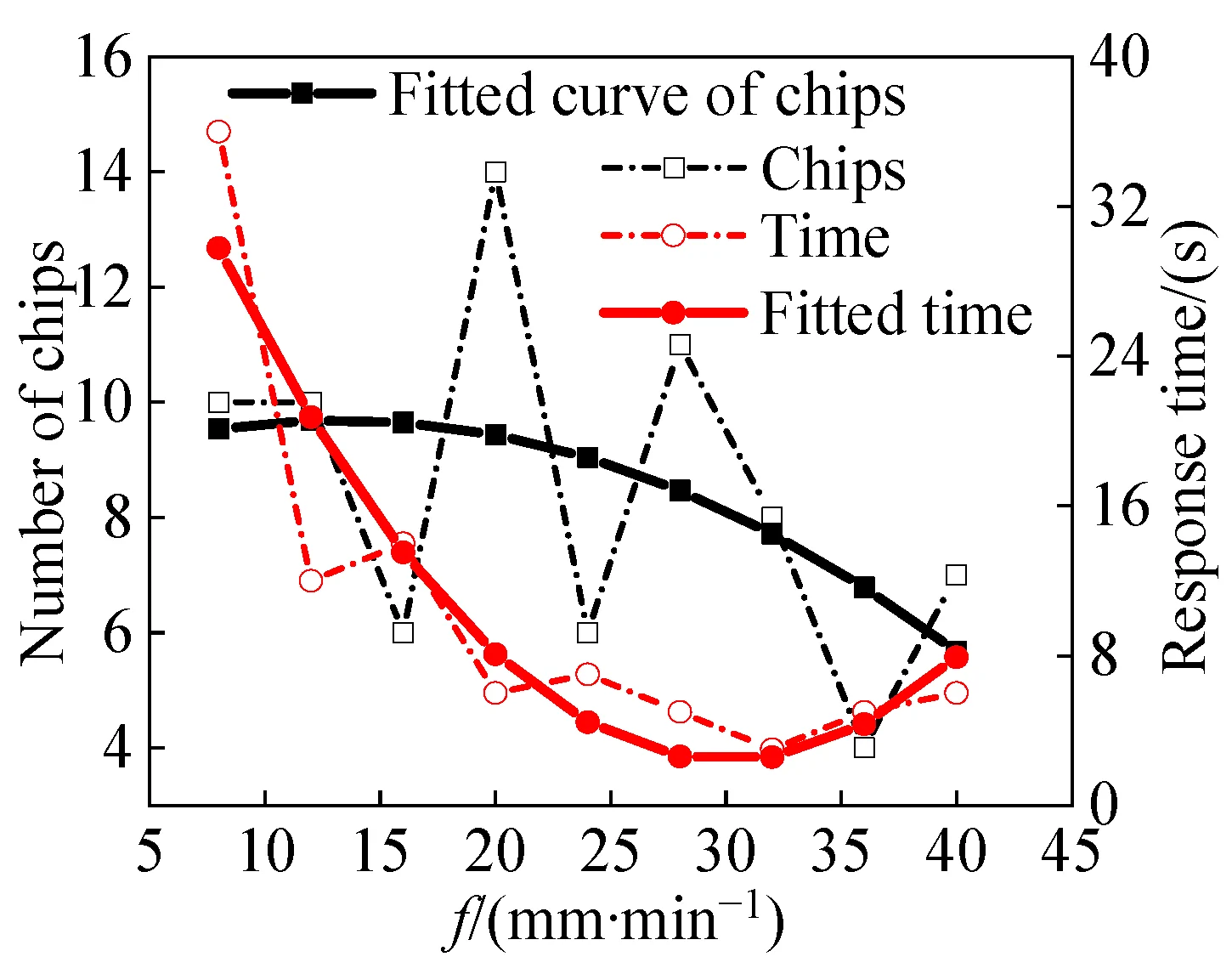
Chip pulse count and time to first chip pulse versus feed rate.

**Figure 18:**
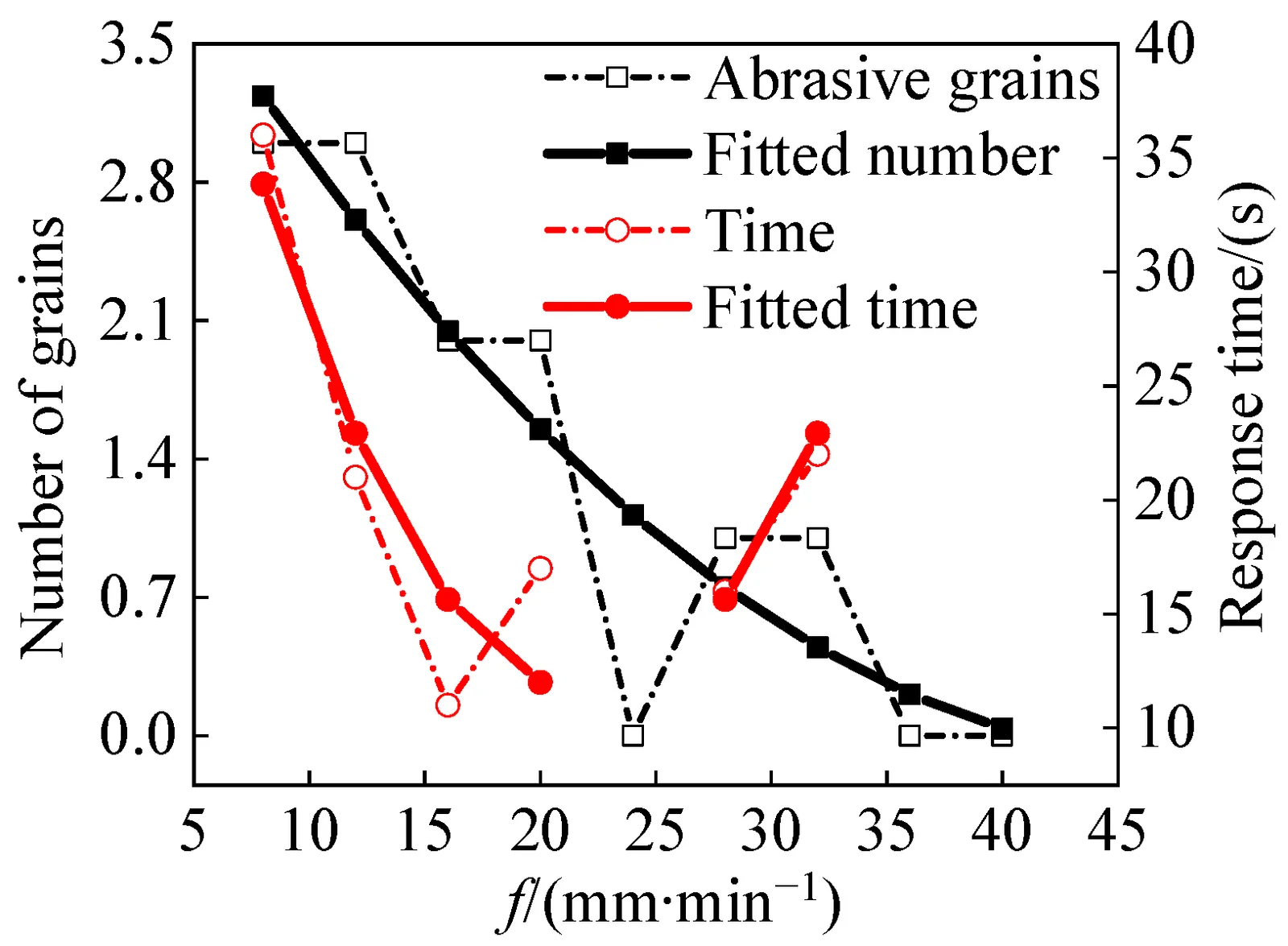
Dislodged-grain pulse count and time to first grain pulse versus feed rate.

Figure 17 and Figure 18 illustrate that rising feed rate generally reduces the pulse counts of grinding chips and detached abrasive grains as well as their corresponding first-pulse delays. This behavior stems from the fixed separation between the electrostatic sensor and the initial grinding zone: newly generated particles need to travel to the sensor’s sensing range to be detected. A higher feed rate cuts this transit duration and shortens the first-pulse delay. Furthermore, faster table travel shortens the grinding duration per unit length, generating fewer chips and lowering the chance of grain detachment. Consequently, both chip and abrasive grain pulse counts decline as feed rate increases.

### 4.4. Monitoring Sensitivity Analysis

Sensor sensitivity is defined as the ratio of the increment in output signal to the increment in input physical parameters, which quantifies the signal variation triggered by unit variations in grinding parameters. Since different sensors yield sensitivity values with distinct physical units, direct numerical comparison cannot judge which sensor achieves superior sensitivity. Accordingly, a dimensionless evaluation metric is required to remove unit interference.

On this basis, the Static Sensitivity Change Degree (SSCD) is proposed to quantify signal fluctuations under different grinding parameters. Taking the sensor signal acquired under reference grinding parameters as the benchmark, SSCD is calculated as the ratio of the measured signal under each altered parameter to this reference signal. The SSCD adopts a fixed reference signal corresponding to the initial machining parameters. Larger parametric increments expand the comparison range and reflect the overall signal evolution across the full parameter interval; nevertheless, excessively large step sizes will mask subtle signal features generated by micro-scale parameter variations.

To address this limitation, this work further proposes the Dynamic Sensitivity Change Degree (DSCD), abbreviated as dynamic sensitivity. A fixed minor parametric step is set as the comparison unit. DSCD is defined as the ratio of the output signal measured after applying the parametric step to the steady-state signal acquired before parameter adjustment.

Prior to DSCD calculation, raw sensor signals undergo standardized sequential preprocessing tailored to working conditions: zero-mean baseline correction, low-pass filtering, and outlier removal, to suppress measurement noise and interference.

Let *S*_k_ denote the steady electrostatic signal under the k-th group of grinding parameters and let *S*_k+1_ represent the measured signal after applying a fixed minor parametric increment. The Dynamic Sensitivity Change Degree (DSCD) is defined as

DSCD_k_ = *S*_k+1_/*S*_k_, (5)

where *S*_k_ and *S*_k+1_ denote the steady-state signal measurements under the k-th and (k + 1)-th test conditions, respectively.

The proposed DSCD index can quantitatively characterize three core sensing performance indicators, as elaborated below:

(1) Local Response Magnitude: Within a single parameter interval, a larger DSCD value indicates a greater relative variation in sensor output under the fixed parametric step.
(2) Signal Stability: Smaller fluctuations in DSCD between consecutive test groups correspond to a more uniform relative growth rate of monitoring signals as grinding parameters change. It should be clarified that a stable growth rate only reflects consistent relative signal variation and does not inherently imply high linearity between input grinding parameters and output sensing signals. The linear fitting quality must be comprehensively assessed via linear correlation coefficients.
(3) Trend of Parametric Variation: A DSCD value greater than 1 indicates that the sensor output rises as grinding parameters increase, while a DSCD value less than 1 corresponds to a declining sensor output. This dimensionless index enables qualitative discrimination of positive or negative correlations between measured electrostatic signals and independent grinding parameters.

#### 4.4.1. DSCD Analysis for Grinding Wheel Speed Monitoring

**Figure 19:**
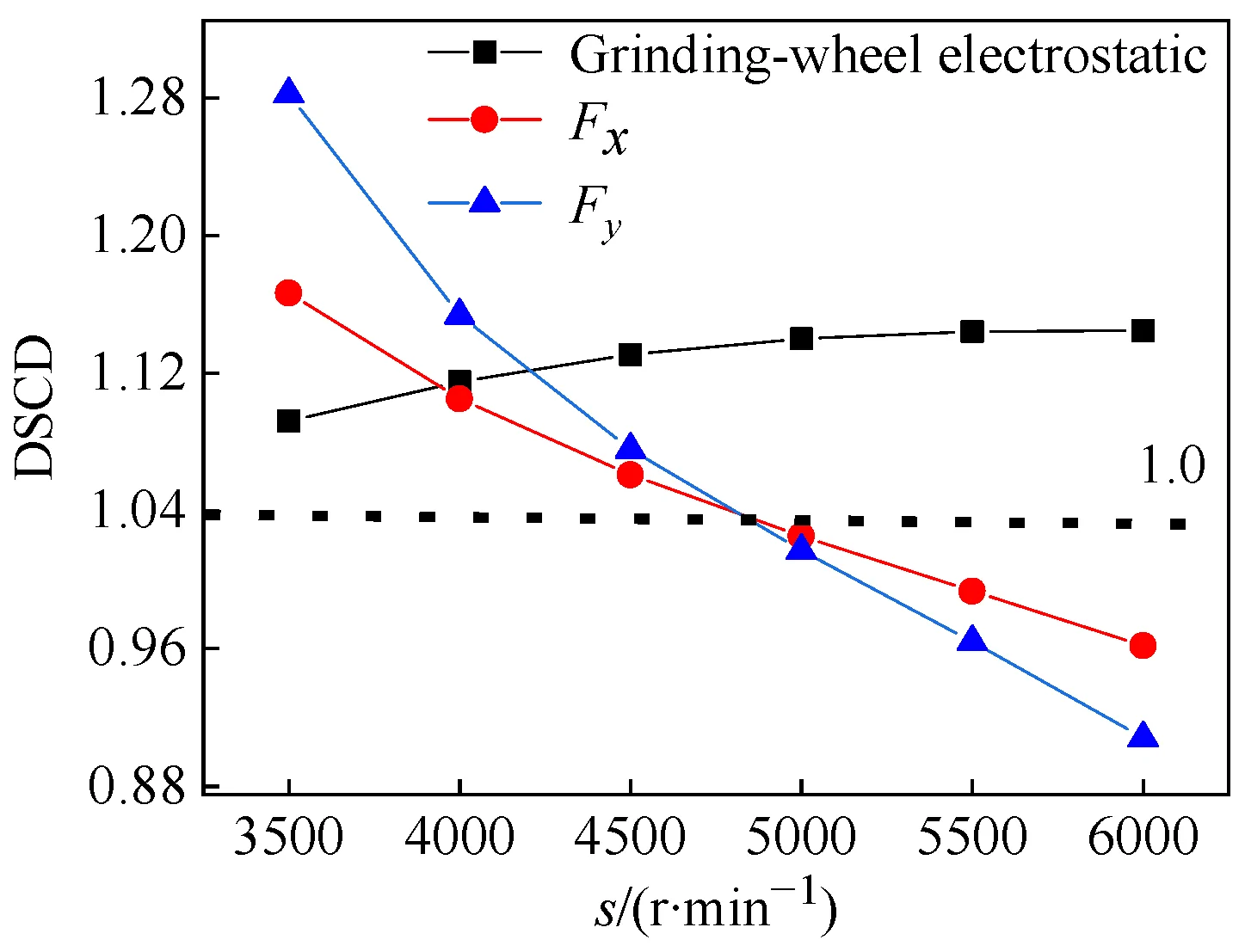
DSCD versus grinding wheel speed.

In the wheel speed variation test, a fixed increment of 500 r·min^−1^ was adopted, and the initial speed of 3000 r·min^−1^ was taken as the reference; the calculated DSCD values are plotted in Figure 19. At low speeds, the electrostatic signal has lower DSCD values than the two force components (normal and tangential force signals). As the wheel speed rises, the DSCD values of normal and tangential force signals decrease. When their values drop below 1, force signals become negatively correlated with wheel speed. Combined with the positive correlation observed at lower speeds, the switching of correlation polarity breaks the monotonic mapping between signal amplitude and wheel speed, hindering continuous quantitative tracking across the full speed range. In comparison, the DSCD of the wheel electrostatic signal remains above 1 and grows monotonically, with a far smoother variation trend than force signals, indicating better stability and correlation performance.

#### 4.4.2. DSCD Analysis for Grinding Depth Monitoring

**Figure 20:**
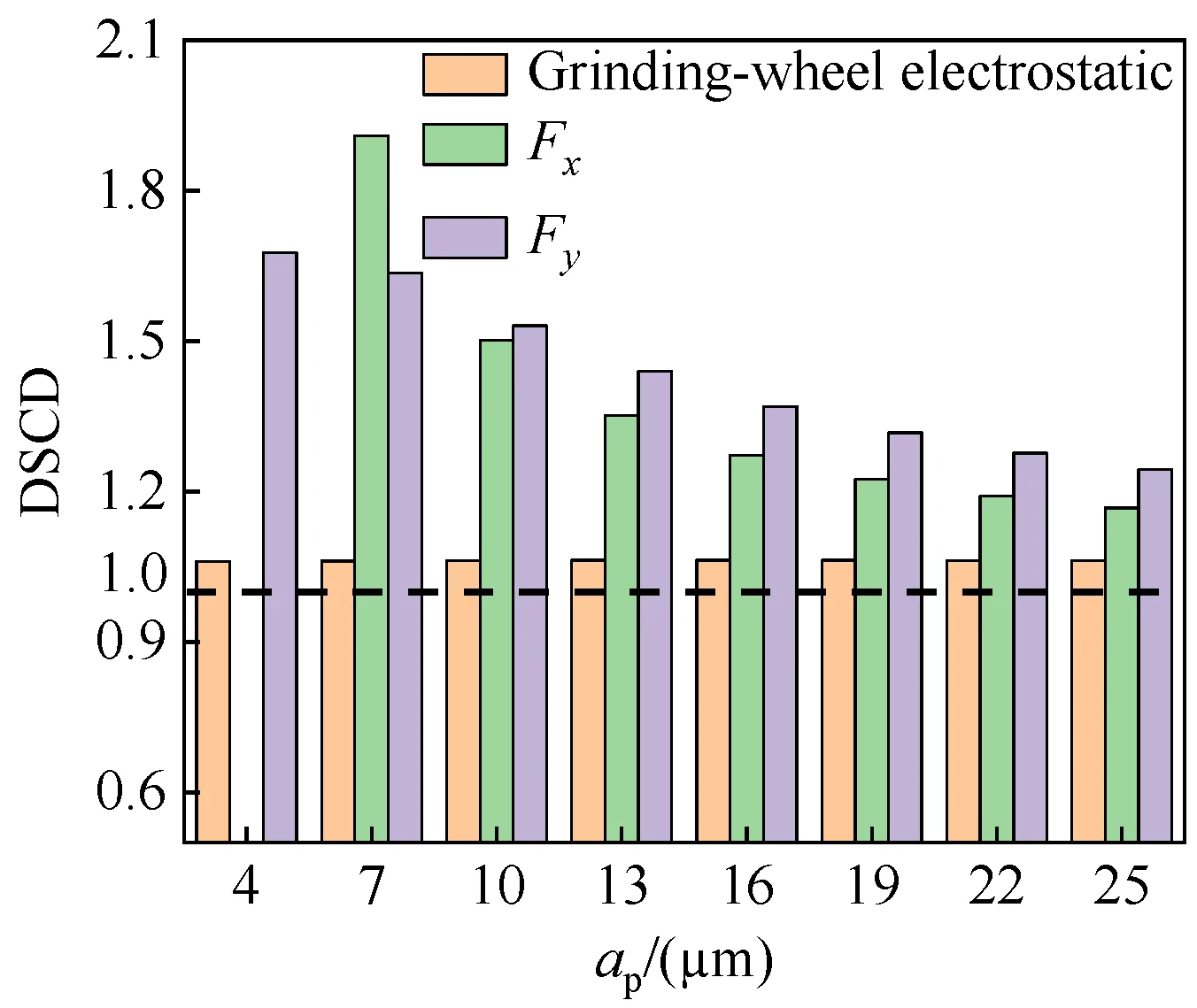
DSCD of monitoring signals versus grinding depth.

A fixed parametric step of 3 μm was adopted, and the initial depth of cut of 1 μm was set as the reference working condition. The DSCD values calculated under each depth of cut are plotted in Figure 20.

**Figure 21:**
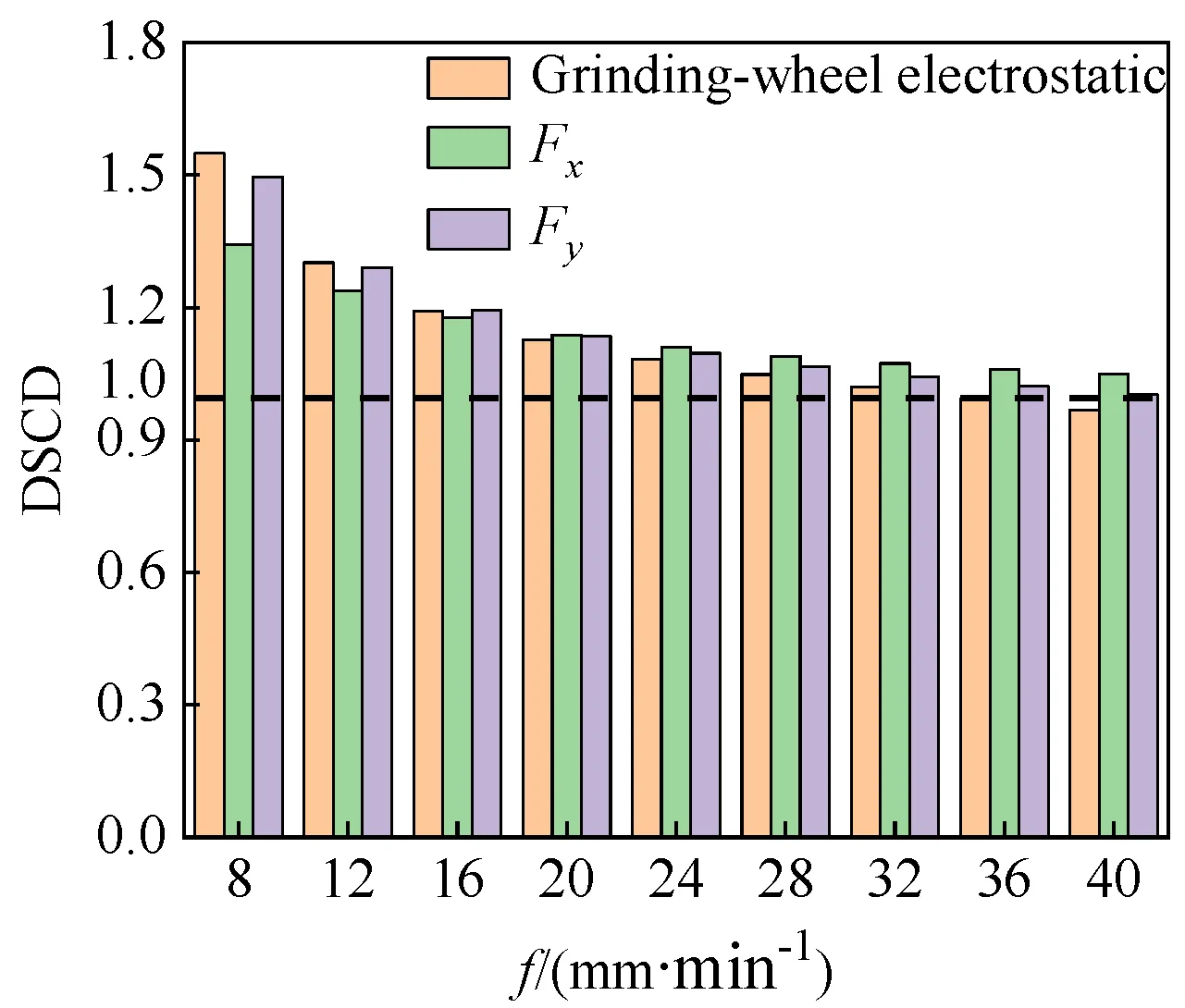
DSCD versus feed rate.

As illustrated in Figure 21, the DSCD of the grinding wheel electrostatic signal is lower than those of both normal and tangential grinding force components. Accordingly, monitoring relying on grinding force yields a more sensitive indicator for tracking variations in depth of cut. Nevertheless, the DSCD curve of the electrostatic signal displays far gentler fluctuations, which verifies that the electrostatic signal possesses a superior linear correlation with depth of cut.

#### 4.4.3. DSCD Analysis for Grinding Feed Rate Monitoring

For the feed rate test, a fixed parametric step of 4 mm·min^−1^ was applied, with an initial feed rate of 1 mm·min^−1^ defined as the reference condition. The corresponding DSCD curves are illustrated in Figure 21. The wheel electrostatic signal, normal grinding force, and tangential grinding force yield DSCD values with highly consistent magnitudes and variation trends. This indicates that when evaluated via the DSCD metric, the force-based monitoring method and the electrostatic monitoring method show negligible performance differences in assessing feed rate variations.

### 4.5. Wheel Wear Monitoring

To reveal the correlation among grinding forces, electrostatic signals and grinding wheel wear, an online wear monitoring test was carried out under the fixed machining parameters summarized in Table 6. The identical workpiece was machined through 40 consecutive grinding passes with a material removal volume of 0.6 mm^3^ per pass, and grinding force and electrostatic signal data were continuously collected throughout the test.

**Figure 22:**
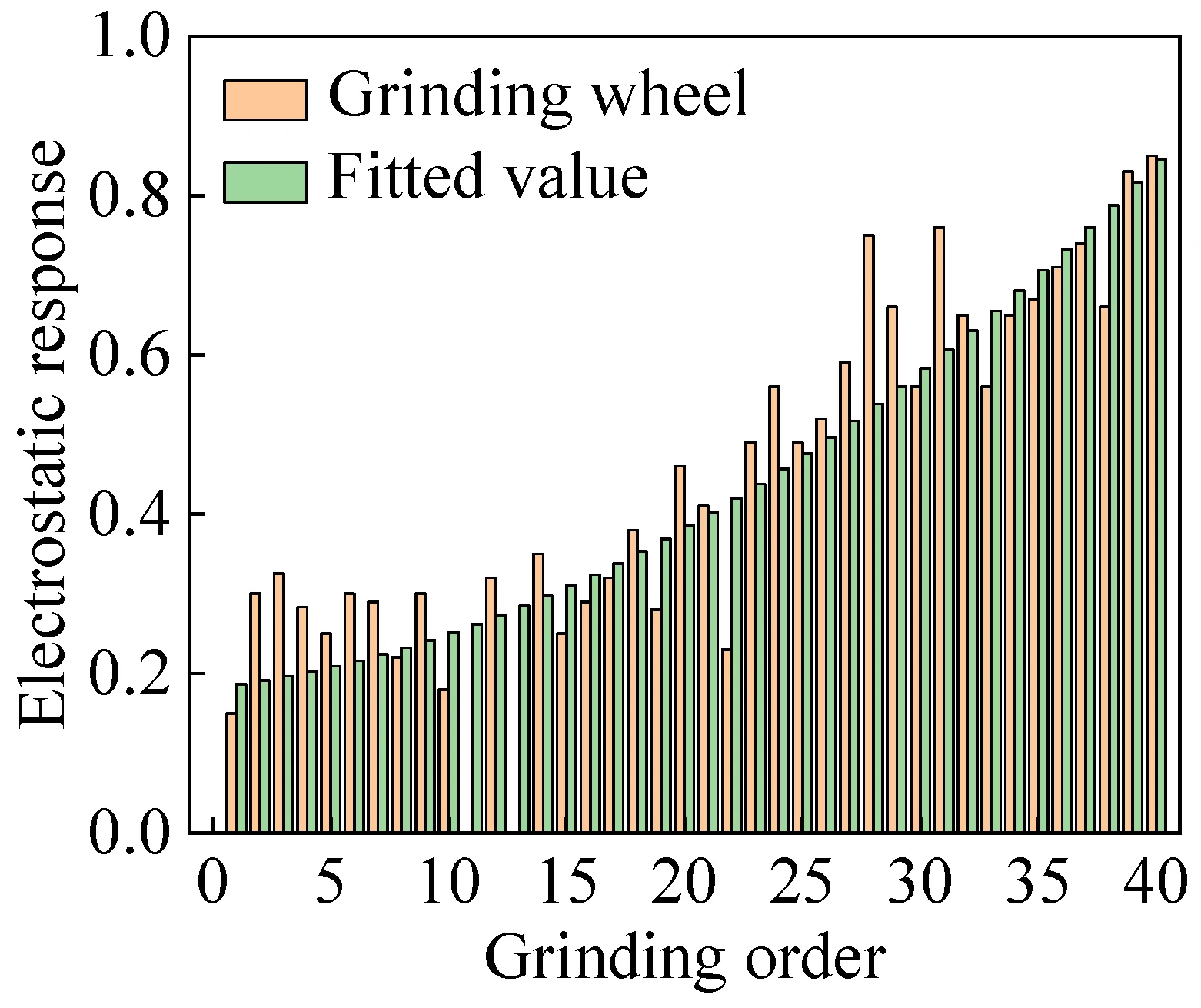
Variation in wheel charge with wear progression.

The wheel electrostatic signal captured throughout the test sequence is illustrated in Figure 22. As the number of grinding passes rises, the accumulated surface charge on the grinding wheel increases monotonically. This reveals that each wear condition corresponds to a unique electrostatic charge level, and the measured signal grows stronger as wheel wear gradually accumulates.

**Figure 23:**
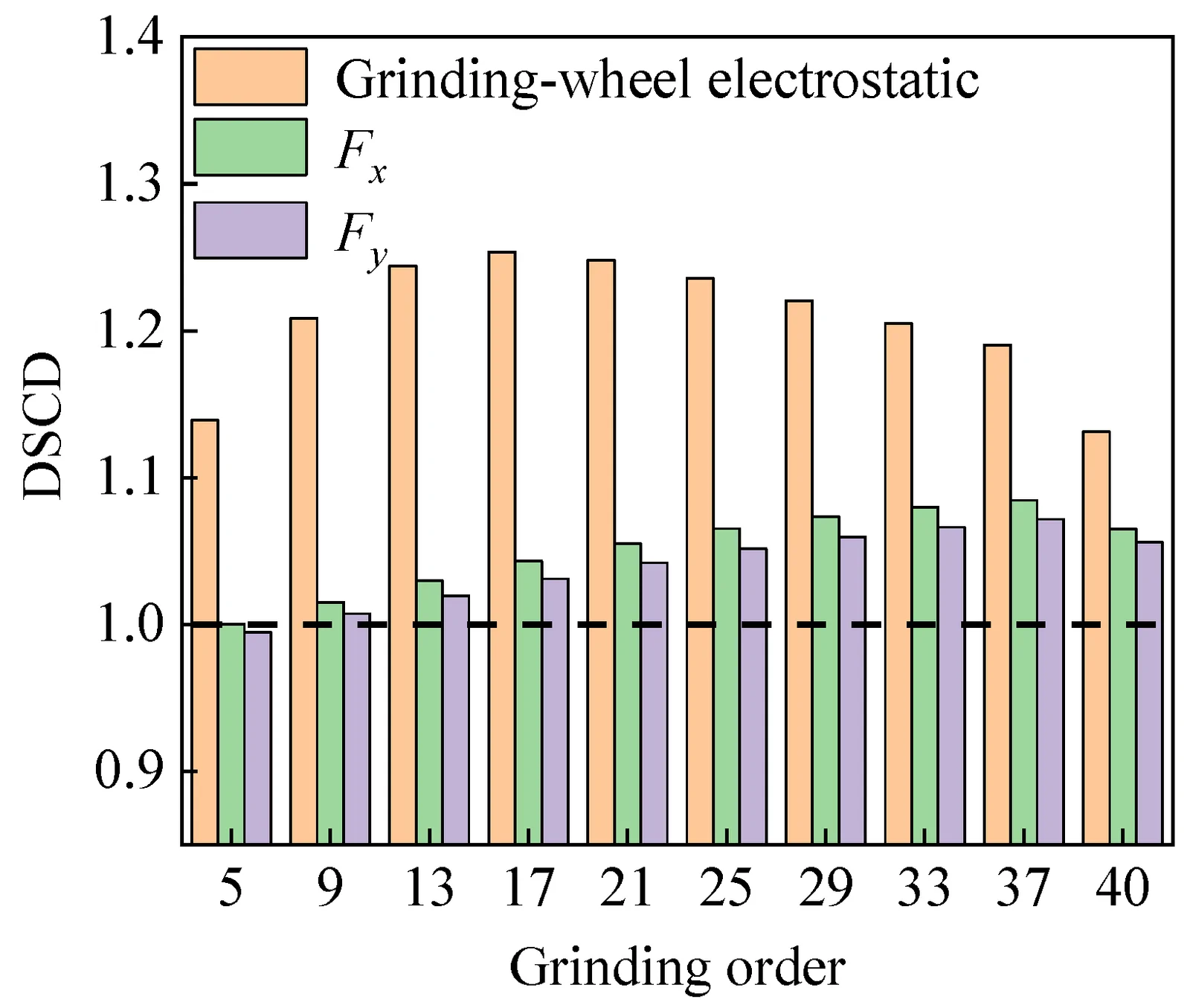
DSCD of the signals during wheel wear progression.

Every four consecutive grinding passes, equivalent to a total material removal volume of 2.4 mm^3^, are defined as one parametric step. The Dynamic Sensitivity Change Degree (DSCD) values of the electrostatic signal, normal grinding force signal, and tangential grinding force signal are calculated accordingly, and the resultant curves are plotted in Figure 23. Over the entire wheel wear evolution process, the electrostatic signal maintains consistently higher DSCD values compared with the two grinding force signals. This confirms that electrostatic sensing exhibits a more sensitive response to the gradual accumulation of grinding wheel wear.

## 5. Data Augmentation Method Based on Electrostatic Signals and Online Evaluation of Grinding Wheel Wear State

### 5.1. Data Augmentation Method Based on Conditional Variational Auto-Encoder

Scarce monitoring data and the inherent black-box property of purely data-driven models are two major bottlenecks restricting the industrial application of machining condition prediction. To address the low interpretability of machine learning models, Wu et al. embedded physical prior knowledge into data-driven model architectures. They further leveraged SHAP and Grad-CAM interpretability tools for real-time cavity prediction in electrochemical machining and constructed a clear correlation between model outputs and underlying machining physical mechanisms [31]. On the other hand, if experimental datasets are insufficient, blind data augmentation without comparative validation tends to degrade the generalization stability of prediction models. Ge et al. systematically evaluated multiple virtual sample generation strategies and explored how the volume of synthetic samples influences prediction accuracy in CFRP drilling state forecasting. Their research demonstrated that data augmentation ought to be treated as a meticulously designed methodological module, rather than a hasty remedy for data scarcity [32]. Drawing on the above two research paradigms, this study proposes a physically interpretable grinding wheel wear diagnosis model. The model incorporates G-CVAE-based virtual sample augmentation for electrostatic grinding condition monitoring. Comparative ablation studies and XAI-driven mechanism analysis are supplemented to enhance the scientific rigor of the proposed method.

**Figure 24:**
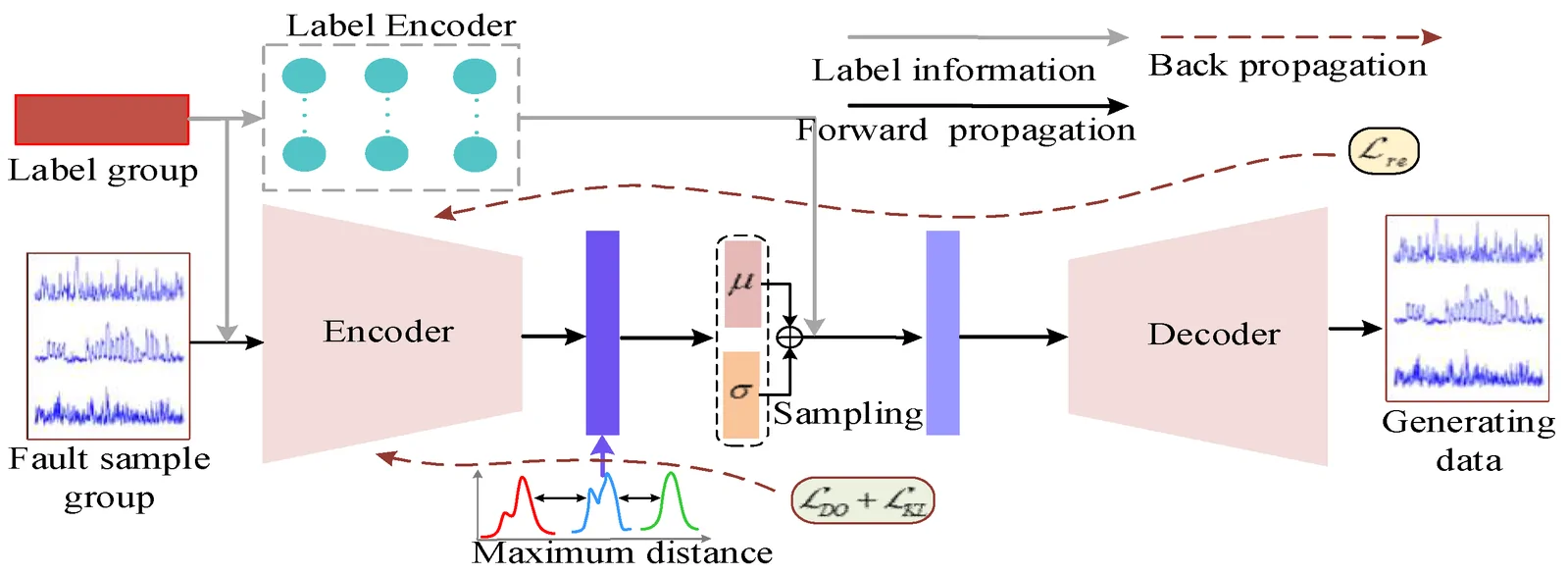
Structural flowchart of the proposed G-CVAE.

This work proposes a Generalized Conditional Variational Auto-Encoder, abbreviated as G-CVAE, whose network architecture is visualized in Figure 24. The model consists of two core sub-networks: (i) an encoder and (ii) a decoder. First, wear state labels are converted into one-hot vectors, which are then explicitly fed into both the encoder and decoder as conditional control inputs.

Two key factors justify the adoption of CVAE as the generative backbone: (1) supervised variational Bayesian inference allows the model to fully utilize label information and learn a discriminative latent distribution corresponding to electrostatic signal features; (2) unlike other mainstream generative models, CVAE supports controllable sample generation. Specifically, synthetic samples can be constrained to a designated wear state, ensuring strict label consistency throughout the data augmentation process.

The specific network architecture is illustrated in Table 7. Linear1_m and Linear1_v correspond to the network structures used to estimate the mean and variance, while Num_class indicates the number of classes.

The encoding network maps the data x_i_ into the feature space through the posterior distribution q(z|x,c), which follows a specific distribution. During the generation process, the type of data generated is controlled by the prior distribution p(x|c), and the decoder reconstructs the data.

### 5.2. Encoding Network

Assuming the original data and labels are ${\left\{x_{i},y_{i}\right\}}_{i=1}^{N}$, and the labels y_i_ are encoded to y_i_^,^, the data and the encoded labels are concatenated to form the input data format $\left[x_{i},y_{i}^{\prime }\right]$, which is then projected by the encoder network $E(:,\theta_{e})$ to obtain the latent vector h_i_, as shown below:

(6) $$h_{i}=E([x_{i},y_{i}^{\prime }];\theta_{e}),$$

The feature vector $h_{i}$ is then passed through two fully connected neural networks to obtain the mean $\vec{\mu }={\left\{\mu^{m}\right\}}_{m=1}^{M}$ and the covariance matrix $\vec{\sigma }={\left\{\sigma^{m}\right\}}_{m=1}^{M}$ of a multivariate Gaussian distribution, where M is the dimensionality of the latent space. The posterior distribution of the corresponding latent variable $z_{i}$ for $x_{i}$ can be described as $q(z_{i}|x_{i},c^{i})=N(z_{i};\vec{u},{\vec{\sigma }}^{2})$. The latent variable $z_{i}$ allows for conditional sampling from multiple models, enabling the model to satisfy a one-to-many mapping construction. At this point, the prior distribution of the generative network for the synthetic data can be expressed as $p(x_{i}|c_{i})=N(x_{i};\vec{\mu },\vec{\sigma })$. To ensure that the distribution of the latent variables matches the prior distribution, the Kullback–Leibler (KL) divergence is used to measure the distribution difference, with the specific loss given by [33]:

(7) $$L_{KL}=-D_{KL}[q(z|x,c)||p(z)]=-\frac{1}{2}\sum_{m=1}^{M}(1+\log({\left(\sigma^{m}\right)}^{2})-{\left(\mu^{m}\right)}^{2}-{\left(\sigma^{m}\right)}^{2}),$$

where p(z): probability density function subject to standard normal distribution N(0,I).

### 5.3. Generation Network

The input to the generation network primarily involves sampling from a multivariate Gaussian distribution $N(\vec{u},{\vec{\sigma }}^{2})$. The sampling process is as follows [33]:

(8) $$z_{i}=\vec{\mu }+\vec{\sigma }\times \delta_{i},$$

where $\delta_{i}$: the data sample randomly collected from the multivariate Gaussian distribution. This sample, combined with the required label information, is passed through the decoder network $D(:,\theta_{d})$ to generate the data sample, with the calculation method as follows:

(9) $$x_{i}^{\prime }=D([z_{i},y_{i}^{\prime }];\theta_{d}),$$

To guarantee that synthetic samples maintain high consistency with raw measured data, this work quantifies data similarity via the mean squared error (MSE) loss between input and reconstructed output samples, defined as

(10) $$L_{\mathrm{Rec}}=\frac{1}{N}\sum_{i=1}^{N}{\left(x_{i}-x_{i}^{\prime }\right)}^{2},$$

where $x_{i}$: input sample; $x_{i}^{\prime }$: generated samples.

Considering the characteristics of the grinding wheel wear data, this paper constructs a G-CVAE with the expectation that there will be significant differences in the distribution of feature space data under different wear states. Generating data with greater variability during the pilot study can provide a better data foundation for subsequent state evaluation. To achieve this functionality, taking into account the differences in the charge carried by the grinding wheel under different states, the hidden features $h_{i}$ extracted by the G-CVAE encoding layer are classified based on the prior information. The hidden features are then categorized into $h^{\prime }={\left\{h_{j}^{c}\right\}}_{j=1}^{n^{\prime }}$, where c is the label type, and $n^{\prime }$ is the number of samples in this category. Based on the label information, multi-dimensional kernel density estimation $f_{c}$ is performed on the data of each state: $f_{c}:=[f(y_{1}^{c}),\ldots ,f(y_{{n^{\prime }}^{c}}^{c})],c=1,\ldots ,M$. The specific expression is as follows:

(11) $$f(y_{i}^{c}):=\frac{1}{nl^{d}}\sum_{j=1}^{{n^{\prime }}^{c}}K(\frac{y_{i}^{c}-h_{j}^{c}}{l}):=\frac{1}{\sqrt{2\pi }nl^{d}}\sum_{j=1}^{{n^{\prime }}^{c}}exp(-\frac{1}{2}{\left(\frac{y_{i}^{c}-h_{j}^{c}}{2}\right)}^{2}),$$

where *i*: serial index of category samples; *n*^′c^: total number of samples in category c; *K*: multivariate Gaussian kernel function; *l*: bandwidth parameter; *y*_c_: sample point to be estimated for category *c*.

The selection of kernel function type has negligible influence on estimation accuracy. The optimal bandwidth of Gaussian kernel follows the formula *l* = (4/(3n))^−1/5^ derived in Ref. [34].

To balance the distribution differences between classes, the maximum mean discrepancy loss is used to evaluate the similarity of the estimated kernel density results.

(12) $$L_{\mathrm{mmd}}={\left\|\left.\sum_{c=1}^{M-1}\sum_{c^{\prime }=c+1}^{M}\left(\frac{1}{{n^{\prime }}^{c}}\sum_{i=1}^{{n^{\prime }}^{c}}\phi (f_{c}(i))-\frac{1}{{n^{\prime }}^{c^{\prime }}}\sum_{j=1}^{{n^{\prime }}^{c^{\prime }}}\phi (f_{c^{\prime }}(j))\right)\right\|\right.}_{F},$$

Thus, the overall loss of the G-CVAE model is given by

(13) $$L_{loss}=L_{KL}+L_{\mathrm{re}}-\lambda L_{\mathrm{mmd}},$$

where λ: the trade-off parameter.

The purpose of the negative $L_{\mathrm{mmd}}$ loss is to increase inter-class separability. To further elaborate the relationship between the encoder and decoder and the above losses, the specific network update process is given by

(14) $$\left\{\begin{array}{l} \theta_{e}\leftarrow \theta_{e}-lr(\frac{\partial L_{KL}+L_{\mathrm{re}}-\lambda L_{\mathrm{mmd}}}{\partial \theta_{e}})\\ \theta_{d}\leftarrow \theta_{d}-lr(\frac{L_{\mathrm{re}}}{\partial \theta_{d}}) \end{array}\right.,$$

where *θ*_e_: the network parameters of the encoder; *θ*_d_: the network parameters of the decoder; *l*_r_: the learning rate of the network. Thus, the loss functions can update each parameter during the training process.

### 5.4. Validation of G-CVAE Effectiveness

Raw monitoring signals contain inherent redundant information, and strong correlations exist between consecutive signal segments. Accordingly, unified preprocessing pipelines are adopted for all collected signals in this study. For each independent signal segment, the first 2500 sampling points (corresponding to a duration of 0.1 s) are extracted to form a single sample, yielding a total of 300 real measured samples. To avoid data leakage caused by random shuffling of highly correlated signal segments from identical grinding passes, dataset partitioning is implemented on the basis of independent grinding passes. More specifically, all samples generated from one grinding pass are fully assigned to either the training set or the test set. This strategy ensures that test samples are derived from unseen grinding processes, enabling an unbiased evaluation of the model’s generalization capability.

Prior to model construction, the dataset is split into disjoint training and test subsets. The test set is exclusively reserved for final performance evaluation and never involved in model training, hyperparameter optimization, preprocessing parameter calculation, or synthetic sample generation. All preprocessing statistics (e.g., mean and standard deviation) are calculated solely from the training set and subsequently applied to the test set. The G-CVAE model only leverages training data to learn latent feature distributions and generate augmented samples; test samples are excluded from model training, synthetic sample screening, and data augmentation workflows. This rigorous data isolation strategy guarantees that test samples correspond to unseen grinding processes, enabling an unbiased assessment of the generalization performance of the proposed G-CVAE under unknown working conditions.

The grinding wheel is regarded as healthy at its initial service stage, labeled State 1. Under this working condition, the wheel surface shows no obvious morphological changes, and the machined workpiece surface remains relatively smooth without prominent scratches or workpiece material adhesion. As the number of grinding cycles accumulates, abrasive grains suffer progressive wear, and pits gradually form on the wheel surface. Once distinct scratches emerge on the machined surface, the wheel is categorized as State 2, corresponding to mild abrasive wear. Continuous operation further aggravates abrasive grain wear, deepens scratches on the machined surface, and induces severe material adhesion. This degradation stage is defined as State 3, representing severe grinding wheel wear.

**Figure 25:**
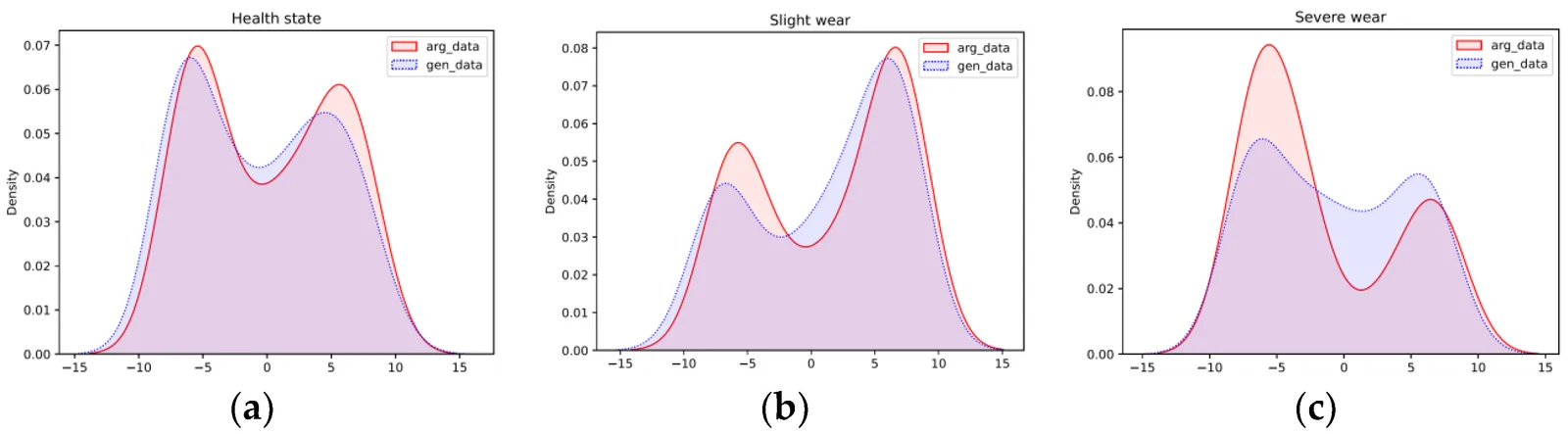
Comparison of distribution between original and generated data for each state: (**a**) healthy state; (**b**) mild wear state; (**c**) severe wear state.

The quality of synthetic samples generated by G-CVAE directly determines the performance of the wear diagnosis model. To verify that the proposed sample generation method accurately learns the marginal distribution features of grinding wheel electrostatic signals, the probability density functions of raw measured data and synthetic samples across all wheel wear stages are plotted in Figure 25. As illustrated, the distribution peaks differ distinctly among the three wear states, laying a solid foundation for subsequent machining condition identification. The synthetic data obtained in this work presents compact distributions that closely match the raw signal distributions, which verifies that the proposed model effectively captures the core distribution characteristics of real electrostatic monitoring signals.

**Figure 26:**
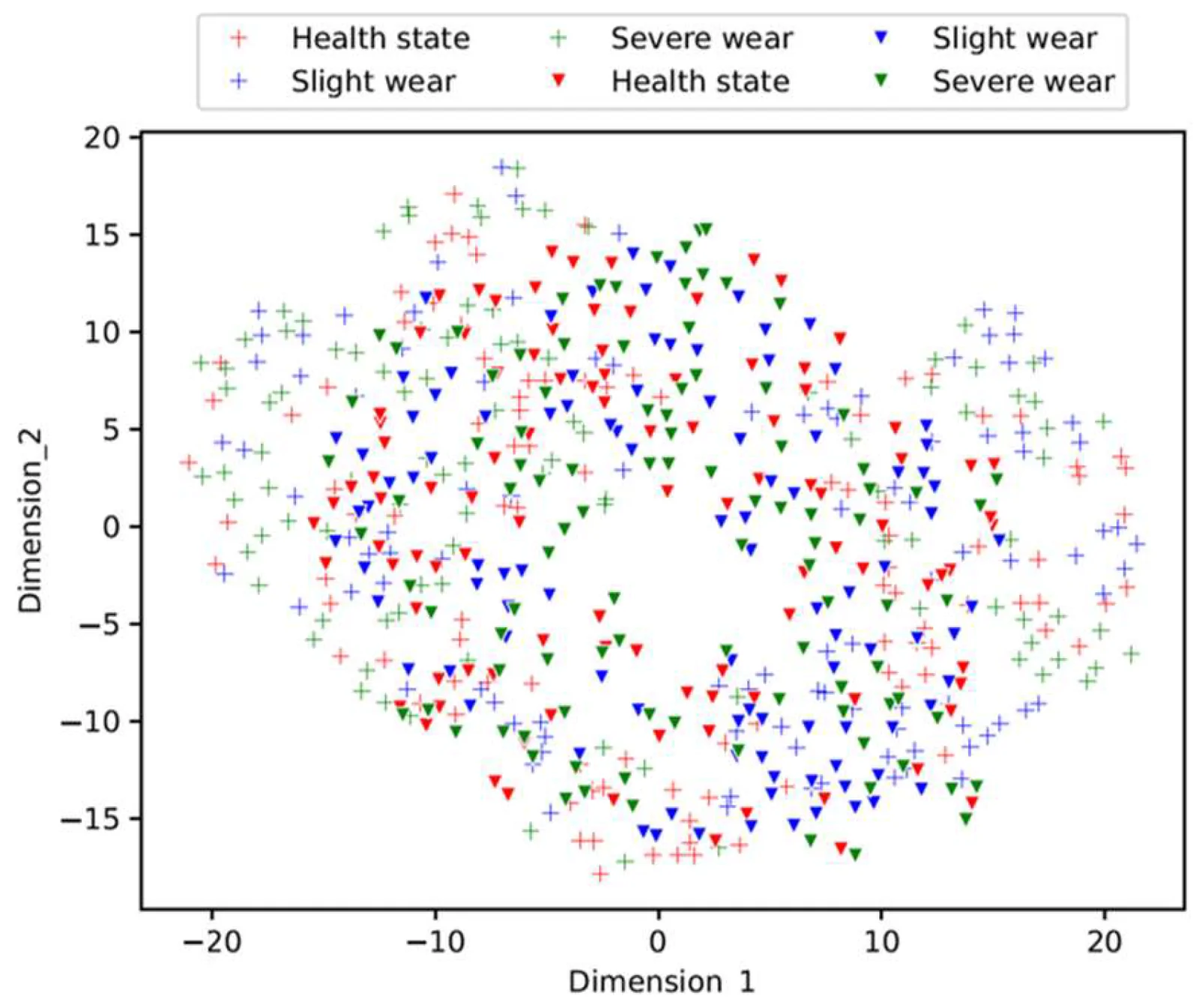
Scatter plots comparing original and generated data.

For an intuitive visual comparison of distribution consistency, high-dimensional signal data are projected into a low-dimensional embedding space via dimensionality reduction. The scatter plots comparing the distributions of raw and synthetic data are displayed in Figure 26. As can be seen from Figure 26, the samples generated by G-CVAE are more compactly clustered while preserving the core distribution features of raw data. Specifically, the cluster centroids corresponding to three wear states (State 1, State 2, State 3) achieve high overlap with those of the original measured data.

**Figure 27:**
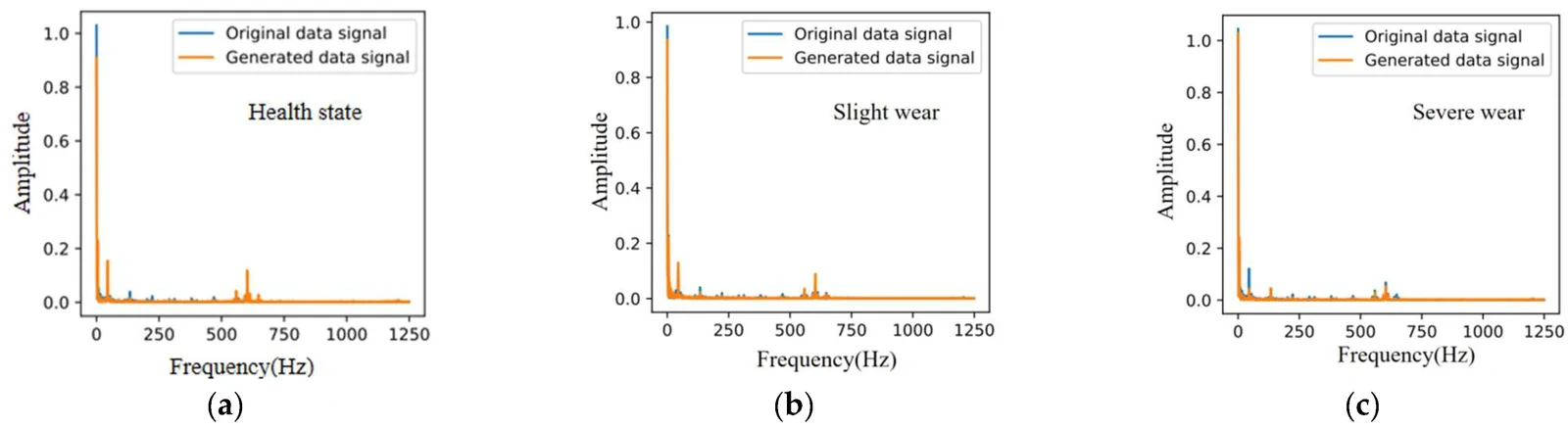
Frequency-domain comparison of generated versus original samples: (**a**) healthy state; (**b**) mild wear state; (**c**) severe wear state.

To further validate the reliability of the proposed sample generation strategy, Fast Fourier Transform (FFT) is adopted to extract and visualize the frequency-domain features of raw measured samples and synthetic samples under all wear states. As shown in Figure 27, the frequency-domain curves of real and synthetic samples exhibit high consistency for each wear condition, with only slight discrepancies observed in a few local frequency bands. This outcome verifies that samples synthesized by G-CVAE simultaneously possess favorable data diversity and stable category invariance across different wheel wear modes.

To quantitatively evaluate the authenticity of samples generated by G-CVAE, maximum mean discrepancy (MMD) and Wasserstein distance are adopted to quantify the distribution discrepancy between measured samples and synthetic samples. Generative Adversarial Network (GAN), Variational Auto-Encoder (VAE) and CVAE are chosen as baseline models for comparison. All evaluation experiments are implemented with an identical sample size configuration (300 measured samples and 300 synthetic samples). The results of various evaluation metrics are summarized in Table 8.

As shown in Table 8, G-CVAE achieves the optimal performance on both metrics. Its MMD value reaches 0.040, which is lower than those of GAN (0.210), VAE (0.280), and CVAE (0.110). The Wasserstein distance of G-CVAE is 1.460, smaller than GAN (2.110), VAE (2.460), and CVAE (1.880). These results reveal that the samples generated by G-CVAE exhibit higher consistency with real grinding vibration signals in the feature space, demonstrating superior capability to capture the statistical distribution characteristics of real data.

It should be noted that a smaller distribution distance merely indicates high statistical similarity between generated and real data and cannot fully reflect sample diversity. The clustering tendency of generated samples in the feature space may enhance category-related features, yet it may also lead to the loss of certain variation patterns. Accordingly, this work comprehensively assesses the effectiveness of data generated by G-CVAE by integrating distribution consistency evaluation, time–frequency domain feature analysis, and performance improvements in downstream classification tasks.

### 5.5. Diagnostic Model Performance Evaluation

To further evaluate whether synthetic data can improve diagnostic accuracy, we construct a diagnostic model following the framework reported in prior research [35]. Separate training and validation experiments are carried out with raw data and generated samples to analyze the performance gain brought by synthetic samples in wear classification tasks.

To verify how virtual samples from diverse generative algorithms affect the diagnostic model, augmented data are separately produced using GAN, VAE, CVAE and the proposed approach. These synthetic samples are fed into the model for training, followed by performance evaluation on real and synthetic test subsets, as listed in Table 9.

Table 9 reveals that every generative algorithm yields moderate performance gains for the diagnostic model, while our proposed method delivers the best comprehensive performance. The overall accuracy (All acc) for GAN, VAE and CVAE is 0.9661, 0.9636 and 0.9708 separately, whereas our method obtains 0.9815, with accuracy increments of 1.54%, 1.79% and 1.07% against the three baseline models. Moreover, our method achieves a real-test-set accuracy (Real acc) of 0.9777, substantially exceeding GAN (0.9618), VAE (0.9588) and CVAE (0.9701). It proves that the synthetic samples generated by our model can significantly enhance the generalization performance for practical grinding monitoring tasks.

In addition, Table 9 shows that our method achieves a Gen acc of 0.9852 on the synthetic test set, exceeding GAN (0.9704), VAE (0.9684) and CVAE (0.9714). This further proves that G-CVAE-generated samples closely match the statistical distribution of real data and retain discriminative features across different grinding states, boosting the feature learning performance of the diagnostic model.

From the comprehensive analysis of distribution consistency and classification accuracy, virtual sample generation is more than just increasing training data quantity. By capturing the latent feature distribution of real grinding signals, it improves the model’s recognition performance for complicated wear modes when training data are scarce. Equipped with class conditional constraints and a variational generative framework, G-CVAE strengthens the expressive power of category features while ensuring high fidelity of generated samples, leading to better data augmentation effects than classic generative algorithms.

To investigate the influence of synthetic sample volume on diagnostic performance, we carry out experiments on G-CVAE with multiple augmentation ratios (real-to-synthetic sample ratios of 1:0, 1:0.5, 1:1, 1:2 and 1:4), and the outcomes are presented in Table 10.

Model accuracy rises at first and gradually stabilizes as more synthetic samples are incorporated. The baseline overall accuracy (All acc) without data augmentation is 0.9687, which climbs to a maximum value of 0.9848 at the ratio of 1:2. At this point, the Real acc and Gen acc achieve 0.9781 and 0.9915 separately, verifying that moderate synthetic samples facilitate the model’s feature extraction. Nevertheless, further expanding the proportion to 1:4 leads to a slight performance drop, as redundant information brought by overmuch generated samples will impair data diversity.

**Figure 28:**
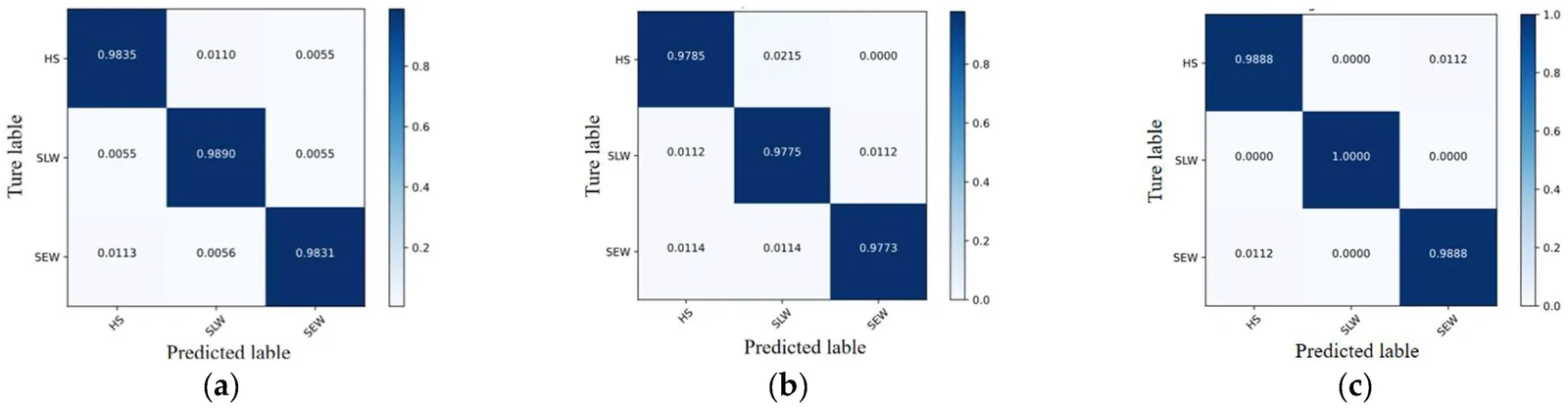
Confusion matrices: (**a**) overall accuracy; (**b**) real sample accuracy; (**c**) generated sample accuracy.

The classification accuracy of the proposed electrostatic signal-based grinding wheel wear diagnosis method is summarized in Table 11, and the corresponding confusion matrix is illustrated in Figure 28. The diagnostic accuracy difference between models trained on real data and synthetic data is negligible, demonstrating that the augmented dataset does not degrade the network training performance.

**Figure 29:**
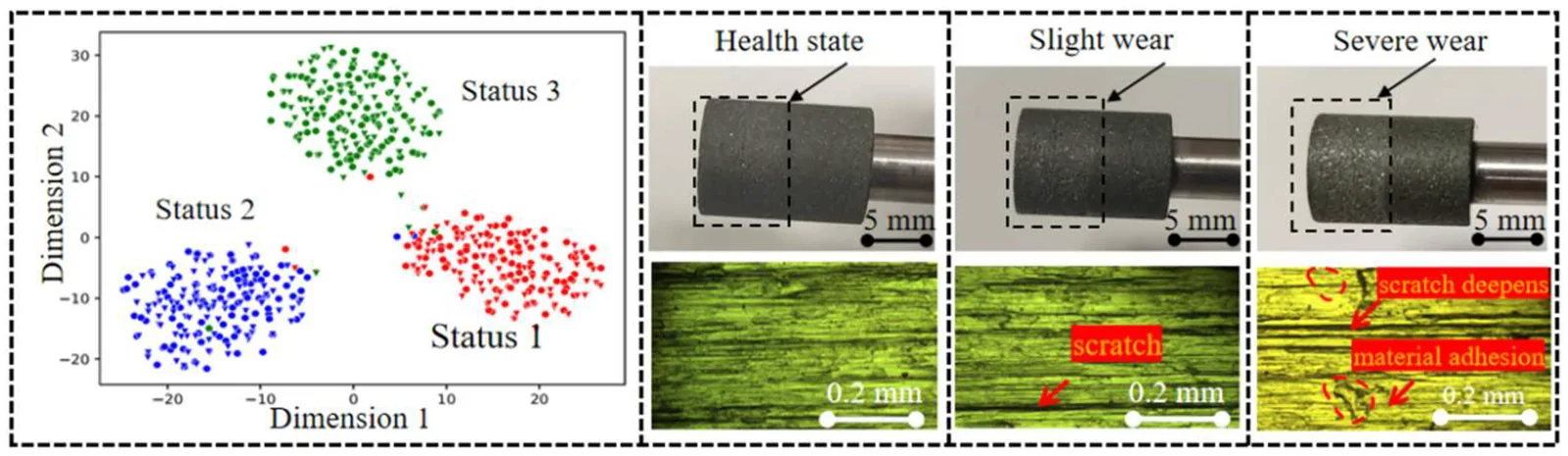
Diagnostic identification under different grinding wheel wear states.

Feature-based classification of electrostatic signals produces the classification outcomes visualized in Figure 29. The three wheel wear states are well-separated in feature space, allowing online identification of corresponding variations in wheel surface topography. This method thus offers an efficient real-time strategy for detecting and distinguishing different grinding wheel wear modes.

## 6. Conclusions

Based on experimental data under varying grinding parameters and wheel wear states, the condensed conclusions are as follows:

(1) The grinding wheel generates electrostatic charge upon workpiece contact, with induced charge increasing monotonically with grinding speed, depth of cut, feed rate and wheel wear degree (charge increments of 106%, 62% and 202%, respectively, under parameter variations). This charge variation enables effective monitoring of grinding parameters and wheel wear conditions.
(2) Grinding chips and dislodged abrasive grains carry opposite electrostatic charges. Pulse counts from chips positively correlate with grinding speed and depth of cut but negatively with feed rate; pulse counts from dislodged grains positively correlate with depth of cut and negatively with feed rate. Time to first pulse for both particles decreases with increasing grinding parameters.
(3) Analysis based on the proposed DSCD index shows that grinding force signals are more applicable to cutting depth monitoring. Electrostatic sensing and grinding force monitoring exhibit equivalent performance when tracking variations in feed rate. In terms of DSCD-based evaluation for monitoring wheel rotational speed and wheel wear conditions, electrostatic signals deliver better detection performance under the present experimental conditions.
(4) The developed G-CVAE model addresses limited wear data by generating high-fidelity synthetic electrostatic signals, effectively expanding datasets and providing robust data support for accurate grinding wheel wear condition assessment.

**Figure 30:**
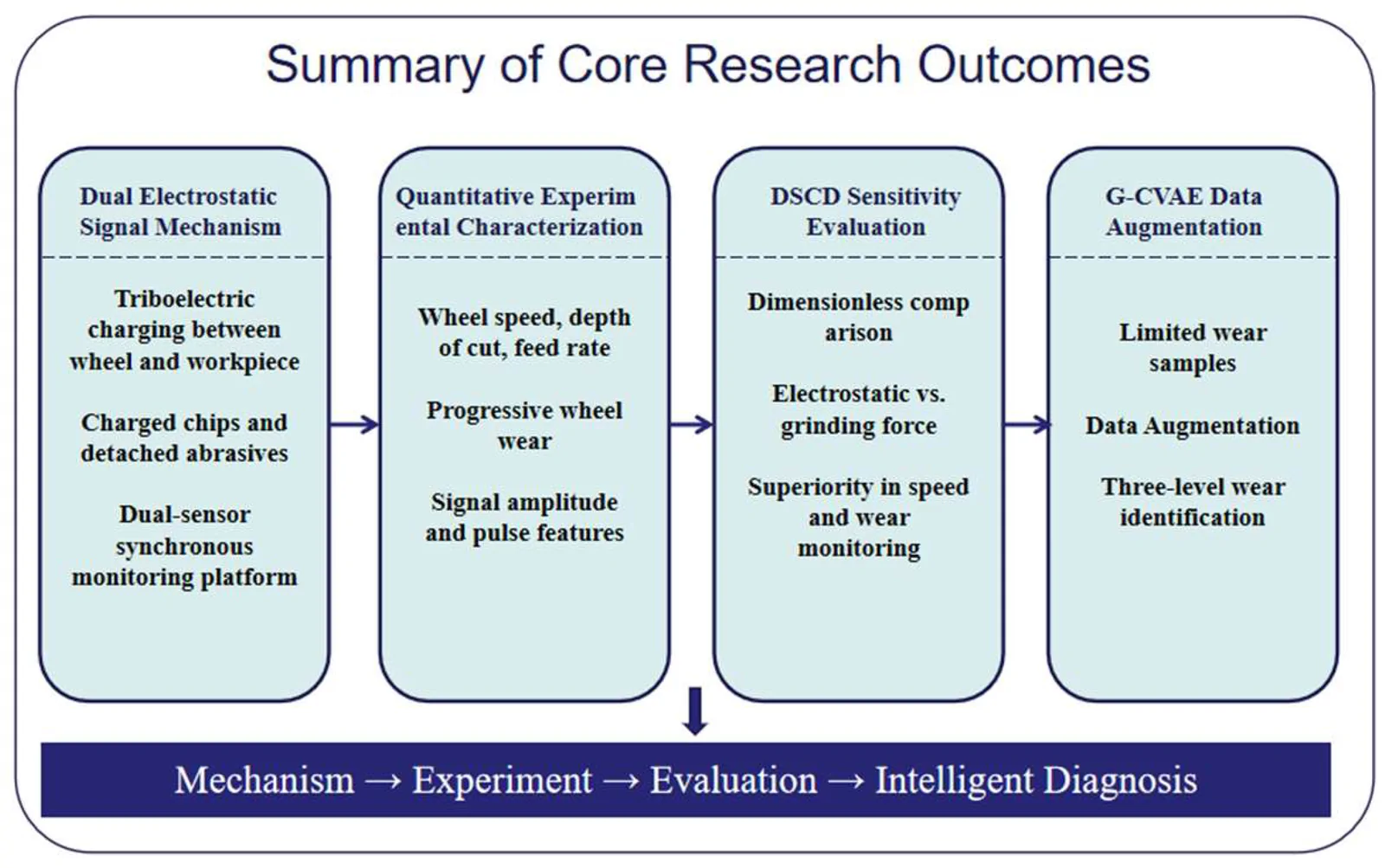
Summary diagram of core research contents of this paper.

**Figure 31:**
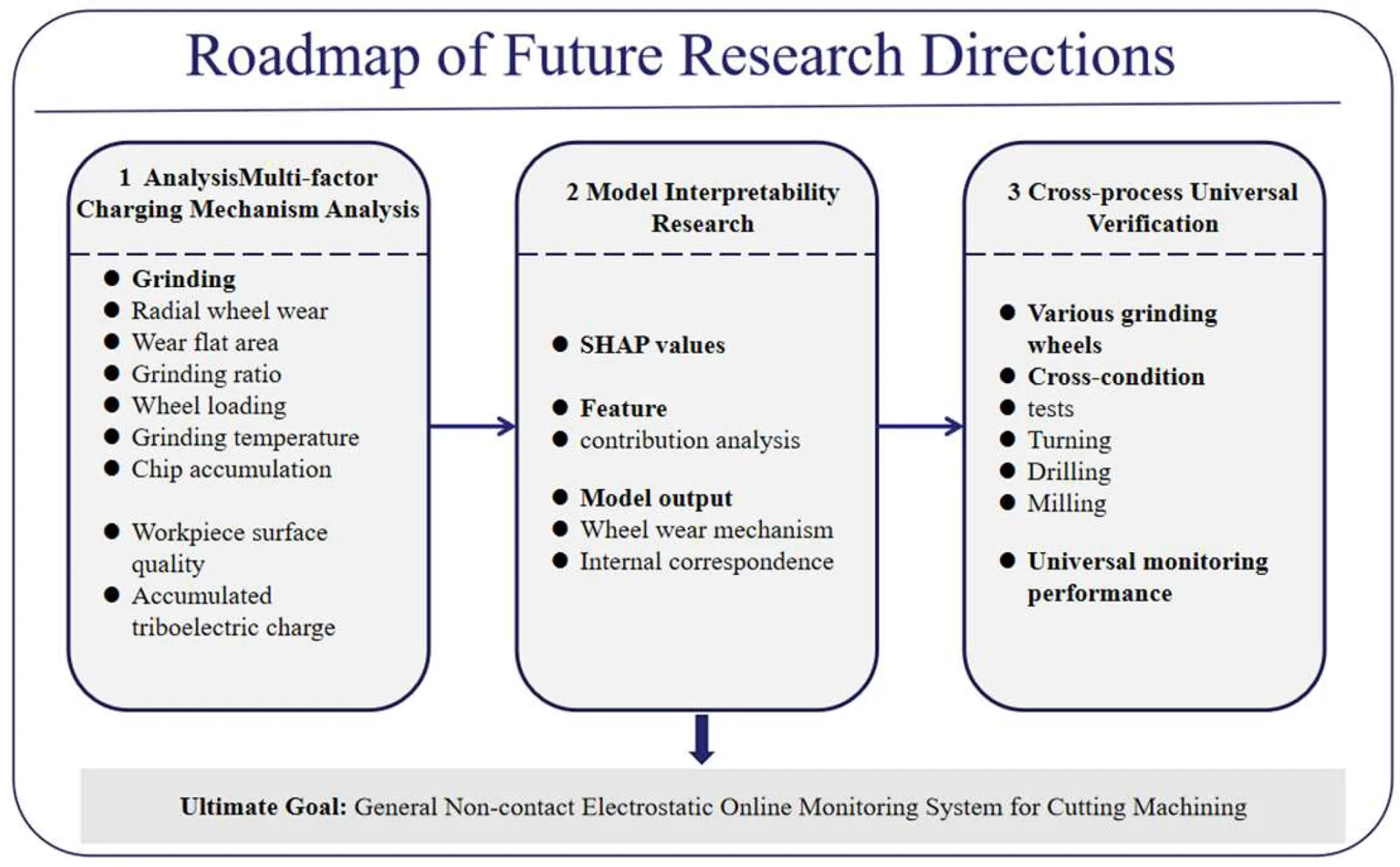
Roadmap of follow-up research directions.

Figure 30 summarizes the overall research content of this study, and Figure 31 illustrates the corresponding future research prospects. Subsequent work will be extended in the following three directions: (1) quantitatively exploring the coupling effects of multiple influencing factors on the variation characteristics of electrostatic signals; (2) investigating the interpretability of the wear diagnosis model to reveal the inherent correlation between model inference results and the physical mechanisms of grinding wheel wear evolution; (3) conducting cross-condition validation experiments with diverse grinding wheels, extending the proposed electrostatic monitoring framework to additional material removal scenarios, and further exploring the universal generalization capability of the presented monitoring method.

## Figures and Tables

**Table 1:** Chemical composition of Ti-6Al-4V alloy (wt%).

Ti-6Al-4V	Al	V	Fe	O	C	N	H	Si
entry 2	6.35	4.1	0.25	0.18	0.06	0.04	0.011	<0.01

**Table 2:** Thermo-mechanical properties of Ti-6Al-4V alloy.

Properties	Value
Density (kg/m^3^)	4429
Elastic modulus E (GPa)	114
Hardness (HV)	340.6
Poisson’s ratio ν	0.33
Yield strength (MPa)	835
Reduction in area (%)	34
Tensile strength (MPa)	905
Thermal conductivity (W/(m·K))	6.7
Melting temperature (°C)	1667

**Table 3:** Experimental parameters at different rotation speeds.

Items	Parameters
Grinding wheel speed, s (r·min^−1^)	3000; 3500; 4000; 4500; 5000; 5500; 6000
Feed rate, f (mm·min^−1^)	15
Depth of grinding, ap (μm)	10

**Table 4:** Experimental parameters at different grinding depths.

Items	Parameters
Grinding wheel speed, s (r·min^−1^)	4000
Feed rate, f (mm·min^−1^)	20
Depth of grinding, ap (μm)	1; 4; 7; 10; 13; 16; 19; 22; 25

**Table 5:** Experimental parameters at different feed rates.

Items	Parameters
Grinding wheel speed, *s* (r·min^−1^)	4000
Feed rate, *f* (mm·min^−1^)	4; 8; 12; 16; 20; 24; 28; 32; 36; 40
Depth of grinding, *a*_p_ (μm)	10

**Table 6:** Grinding parameters.

Items	Parameters
Grinding wheel speed, *s* (r·min^−1^)	4000
Feed rate, *f* (mm·min^−1^)	15
Depth of grinding, *a*_p_ (μm)	10

**Table 7:** G-CVAE network structure.

Networks	Layers	Operations			
		Kernel Size	Stride	BN	Activation
Encoder	Conv1d	5	1	True	Relu
	Conv1d	5	1	True	Relu
	MaxPool1d	3	2	-	-
	Linear1_m	1024	-	-	Relu
	Linear2_m	512	-	-	Relu
	Linear3_m	32	-	-	-
	Linear1_v	1024	-	-	Relu
	Linear2_v	512	-	-	Relu
	Linear3_v	32	-	-	-
Decoder	Linear1	256	-	True	Relu
	Linear2	512	-	True	Relu
	Linear3	2500	-	True	-
Class net	Linear1	512	-	True	Relu
	Linear1	64	-	True	Relu
	Linear1	Num_class = 3	-	True	softmax

**Table 8:** Evaluation of distribution consistency between real samples and samples generated by different generative methods.

Methods	Sample Size	MMD	Wasserstein Distance
GAN	300 vs. 300	0.21	2.11
VAE		0.28	2.46
CVAE		0.11	1.88
Our method		0.04	1.46

**Table 9:** Effects of data augmentation with various generative methods on diagnostic model performance.

Methods	All_Acc	Real_Acc	Gen_Acc
GAN	0.9661	0.9618	0.9704
VAE	0.9636	0.9588	0.9684
CVAE	0.9708	0.9701	0.9714
Our method	0.9815	0.9777	0.9852

**Table 10:** Effects of the quantity of synthetic samples on diagnostic model performance.

Methods	Ratio	All_Acc	Real_Acc	Gen_Acc
Our method	1:0	0.9687	0.9687	-
	1:0.5	0.9740	0.9709	0.9771
	1:1	0.9815	0.9777	0.9852
	1:2	0.9848	0.9781	0.9915
	1:4	0.9827	0.9725	0.9929

**Table 11:** Comparison of detection accuracy between generated and real data.

Method	Accuracy
All data	0.9852
Real data only	0.9778
Generated data only	0.9925

## Data Availability

The data presented in this study are available on request from the corresponding author.

## Abbreviations

The following abbreviations are used in this manuscript:

CSCD	Static Sensitivity Change Degree
DSCD	Dynamic Sensitivity Change Degree
G-CVAE	Generalized Conditional Variational Auto-Encoder
GAN	Generative Adversarial Network
VAE	Variational Auto-Encoder
CVAE	Conditional Variational Auto-Encoder
AE	Acoustic Emission
EMI	Electromechanical impedance
CBN	Cubic Boron Nitride
MSD	Maximum Mean Discrepancy
